# Cretaceous amber inclusions illuminate the evolutionary origin of tardigrades

**DOI:** 10.1038/s42003-024-06643-2

**Published:** 2024-08-06

**Authors:** Marc A. Mapalo, Joanna M. Wolfe, Javier Ortega-Hernández

**Affiliations:** Museum of Comparative Zoology and Department of Organismic and Evolutionary Biology, Harvard University, Cambridge, MA USA

**Keywords:** Palaeontology, Phylogenetics, Taxonomy

## Abstract

Tardigrades are a diverse phylum of microscopic invertebrates widely known for their extreme survival capabilities. Molecular clocks suggest that tardigrades diverged from other panarthropods before the Cambrian, but their fossil record is extremely sparse. Only the fossil tardigrades *Milnesium swolenskyi* (Late Cretaceous) and *Paradoryphoribius chronocaribbeus* (Miocene) have resolved taxonomic positions, restricting the availability of calibration points for estimating for the origin of this phylum. Here, we revise two crown-group tardigrades from Canadian Cretaceous-aged amber using confocal fluorescence microscopy, revealing critical morphological characters that resolve their taxonomic positions. Formal morphological redescription of *Beorn leggi* reveals that it features *Hypsibius*-type claws. We also describe *Aerobius dactylus* gen. et sp. nov. based on its unique combination of claw characters. Phylogenetic analyses indicate that *Beo. leggi* and *Aer. dactylus* belong to the eutardigrade superfamily Hypsibioidea, adding a critical fossil calibration point to investigate tardigrade origins. Our molecular clock estimates suggest an early Paleozoic diversification of crown-group Tardigrada and highlight the importance of *Beo. leggi* as a calibration point that directly impacts estimates of shallow nodes. Our results suggest that independent terrestrialization of eutardigrades and heterotardigrades occurred around the end-Carboniferous and Lower Jurassic, respectively. These estimates also provide minimum ages for convergent acquisition of cryptobiosis.

## Abstract (web summary)

The redescription of two Cretaceous-aged tardigrade fossils resolved their relationship with modern species, with one belonging to a new genus. This new information is critical for calibrating the timing of the evolutionary origins of tardigrades.

## Introduction

Tardigrades are microscopic invertebrates characterized by a compact body plan with four pairs of typically claw-bearing lobopodous legs^1^ that are closely related to onychophorans and euarthropods as members of Panarthropoda^2^. Tardigrades are popularly known for the cryptobiotic ability of some species that allow them to survive extreme conditions, such as space vacuum, ionizing radiation, and low subzero temperatures^3^, as well as their worldwide distribution in marine, freshwater, and terrestrial habitats^4^. Despite their ubiquitous nature in the present-day biosphere, tardigrades have a notoriously scarce fossil record, which limits the study of their macroevolution including the origin of their body plan, and the timing of their terrestrialization and acquisition of cryptobiotic capabilities. Currently, there are only four known crown-group tardigrade fossils, all of which are preserved as amber inclusions^5–7^, but only two of them have well-established taxonomic positions relative to extant tardigrades.

The stratigraphically oldest known crown-group tardigrade fossil is *Milnesium swolenskyi* (*Mil. swolenskyi*)^6^, found in New Jersey (Raritan) amber and dated to the Turonian Age in the Cretaceous Period (89.8–93.9 Mya). The presence of cephalic papillae and *Milnesium*-type claws resolve *Mil. swolenskyi* (three-letter abbreviations of genera used according to refs. ^8,9^) within the extant family Milnesiidae (order Apochela). Given the lack of distinguishable external morphological characteristics between *Mil. swolenskyi* and its modern relatives, this taxon has been considered as evidence for morphological stasis for at least 90 million years^6^. *Paradoryphoribius chronocaribbeus* (*Par. chronocaribbeus*)^7^ is the stratigraphically youngest, and most recently described, fossil tardigrade, and it is embedded in Dominican amber and dated in the Miocene (~16 Mya). *Par. chronocaribbeus* shows some morphological differences compared to extant tardigrades in terms of its internal buccal apparatus, but the presence of typical *Isohypsibius*-type claws supports its placement among the superfamily Isohypsibioidea, order Parachela^7^.

The affinities and morphology of the remaining fossil tardigrades are less clear. These two specimens represent the first case of tardigrades discovered in the fossil record and are both embedded in the same piece of Canadian amber dated to the Campanian Age in the Cretaceous Period (72.1–83.6 Mya)^5^. *Beorn leggi* (*Beo. leggi*)^5^, the first fossil tardigrade ever discovered and named, is generally regarded to have a eutardigrade-like body, but its exact position within the class is still unknown because the description of important taxonomic characters remains vague, particularly the morphology of the claws. For example, it is uncertain whether the claws in *Beo. leggi* are divided or joined, which is an important distinction for determining the higher-level affinity among eutardigrades. Although initially regarded as a monospecific taxon in the completely extinct family Beorniidae^5^, it has been more recently suggested that *Beo. leggi* might belong to an extant family, such as Isohypsibiidae and Murrayidae^6,10^. However, the detailed morphology and thus precise affinities of *Beo. leggi* remain problematic. The second tardigrade within the same piece of Canadian amber identified by Cooper^5^ was deemed to be too poorly preserved to allow proper identification, but a heterotardigrade affinity was suggested based on the presence of filiform structures interpreted as lateral cirri and clavae. With the advancement of imaging techniques and current revisions to tardigrade taxonomy, formal redescriptions of these two fossils can help illuminate the evolutionary history of this major animal clade, and better constrain the time of its origination based on additional fossil calibration points for molecular dating.

The first study that involved molecular dating of tardigrade clades was done using three protein-coding genes and estimated a Precambrian diversification of crown-group tardigrades between the late Cryogenian and early Ediacaran (627–691 Mya^11^). Nearly a decade later, a study mainly focusing on ecdysozoans and including four eutardigrades was done^12^. The results provide the first estimate on the divergence of eutardigrades (i.e., split of apochelans and parachelans), around the Carboniferous. However, this study used the putative tardigrade fossil from the Siberian Orsten^13,14^. This fossil lacks characters defining extant tardigrades, and therefore can only be regarded as part of the stem group^10^. Later studies focused on specific tardigrade groups, such as echiniscids^15^ and milnesiids^16^. Both studies estimated the split of crown-group tardigrades around the Ediacaran, but only the milnesiid-focused study used an uncontroversial tardigrade fossil, *Mil. swolenskyi*, as a calibration. The latest ecdysozoan-focused molecular dating also used *Mil. swolenskyi* to calibrate the crown-group tardigrade while mentioning that *Beo. leggi* is considerably younger^2^. Unlike previous studies, their results showed that the crown-group tardigrades diversified at the start of the Paleozoic, around the late Cambrian. None of these studies have incorporated *Beo. leggi* as a fossil calibration because of its younger age, and the fact that the precise affinities of this taxon have not been formally revised for over 50 years.

In this study, we produced high-quality confocal fluorescence microscopy images of the external morphology of *Beo. leggi* and the undescribed second tardigrade from Canadian amber to resolve their phylogenetic affinities. We use the new data on the morphology and affinities of these fossils to inform the timing of tardigrade origins based on new molecular clock estimates that include a comprehensive sample of extant tardigrade diversity.

## Results

### Morphological redescription of *Beorn leggi*

Confocal fluorescence microscopy allowed us to obtain high-resolution images of the amber inclusions that reveal taxonomically significant features. See Supplementary Information for detailed Systematic Paleontology (Supplementary Text, measurements in Tables S4 and S5, Fig. S1).

**Fig. 1:**
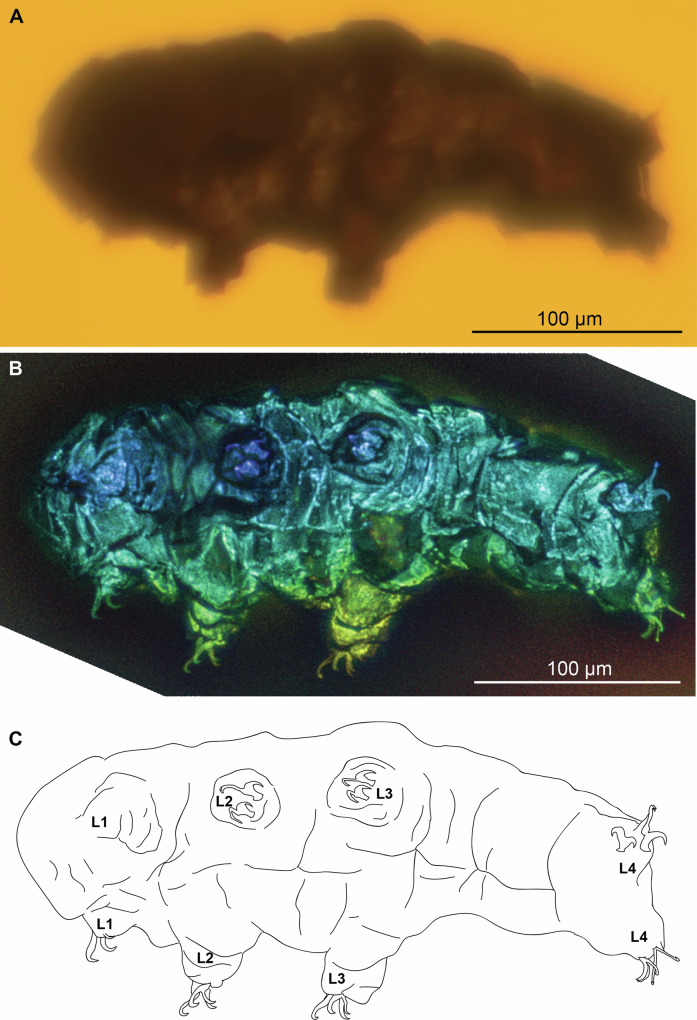
Ventral view of *Beorn leggi* (MCZ PALE-5213). **A** Specimen photographed with transmitted light under compound microscope. **B** Specimen photographed with autofluorescence under confocal microscope at 639 nm; different colors indicate *z*-depth, with violet to red gradient representing the shallowest to deepest planes, respectively. **C** Schematic drawing. L*n* leg number.

The holotype (MCZ PALE-5213) and only known specimen of *Beorn leggi* is a complete body fossil that is clearly visible in dorso-ventral view (Figs. 1, S2A, S3A, B). The individual has a length of at least 309 μm since it is slightly bent at the section between the third and fourth pairs of legs. The cuticle appears to be smooth with no visible cuticular extensions, but cuticular folds are expressed on the dorsal side, potentially produced during preservation. Eyespots were not observed. It was not possible to visualize the mouth opening and buccal apparatus to determine their morphology through transmitted light nor confocal fluorescence.

**Fig. 2:**
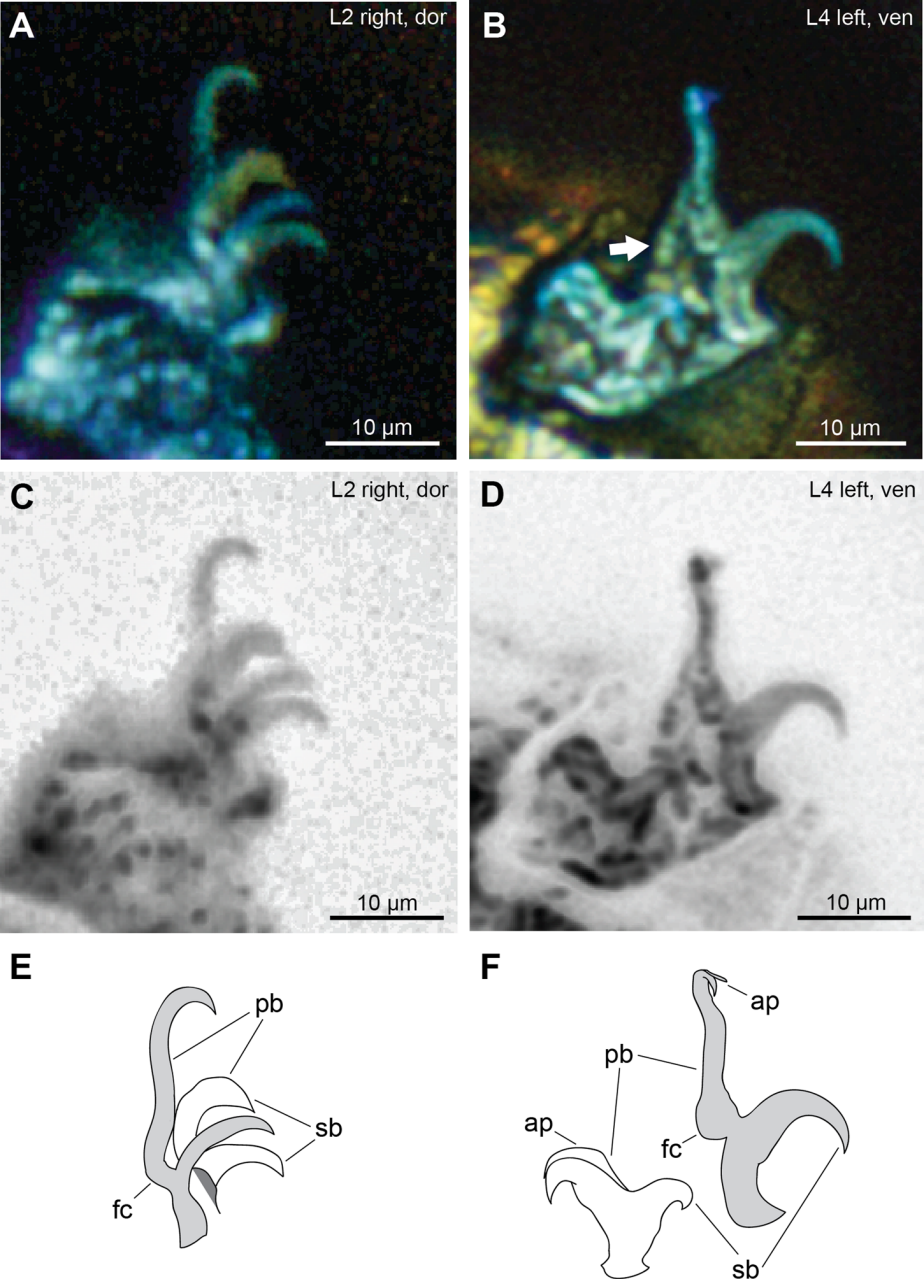
Claws of *Beorn leggi* (MCZ PALE-5213). **A**, **B** Structures photographed with autofluorescence under confocal microscope at 639 nm; different colors indicate *z*-depth, with violet to red gradient representing the shallowest to deepest planes, respectively; arrow indicates cuticular extension. **C**, **D** Claws viewed in inverted greyscale to highlight autofluorescence intensity (darker—more intense, lighter—least intense). **E**, **F** Schematic drawing. Light gray shade—external (legs II) and posterior claws (leg IV); unshaded—internal (leg II) and anterior claws (leg IV); dark gray shade—leg portion. ap accessory points, dor dorsal view, fc flexible connection, L*n* leg number, pb primary branch, sb secondary branch, ven ventral view.

The legs of MCZ PALE-5213 are lobopodous but feature transverse cuticular folds likely produced by cuticle shrinkage during preservation, and it is not telescopic as indicated in its original description. All claws are visible and well-preserved, except on the first left leg (Figs. 1, 2, S2B–M, S3C–J), most likely caused by either loss of the claw during preservation or the inability to detect it through microscopy. The external and internal claws (posterior and anterior claws in the fourth leg) differ greatly in shape and size (Fig. 2). The external and posterior claws feature a secondary branch forming a continuous curve with its basal tract and the primary branch connected with an evident flexible part while the internal and anterior claws are more robust and rigid (Fig. 2E, F). These features correspond to *Hypsibius*-type claws^17^ as expressed in members of the family Hypsibiidae^18^. The external and posterior claws have primary branches that are clearly longer than the secondary branches (Table S4). Pseudolunules were not observed, while accessory points are observed on the posterior claw (Fig. 2F).

### Morphological description of *Aerobius dactylus* gen. et sp. nov

**Fig. 3:**
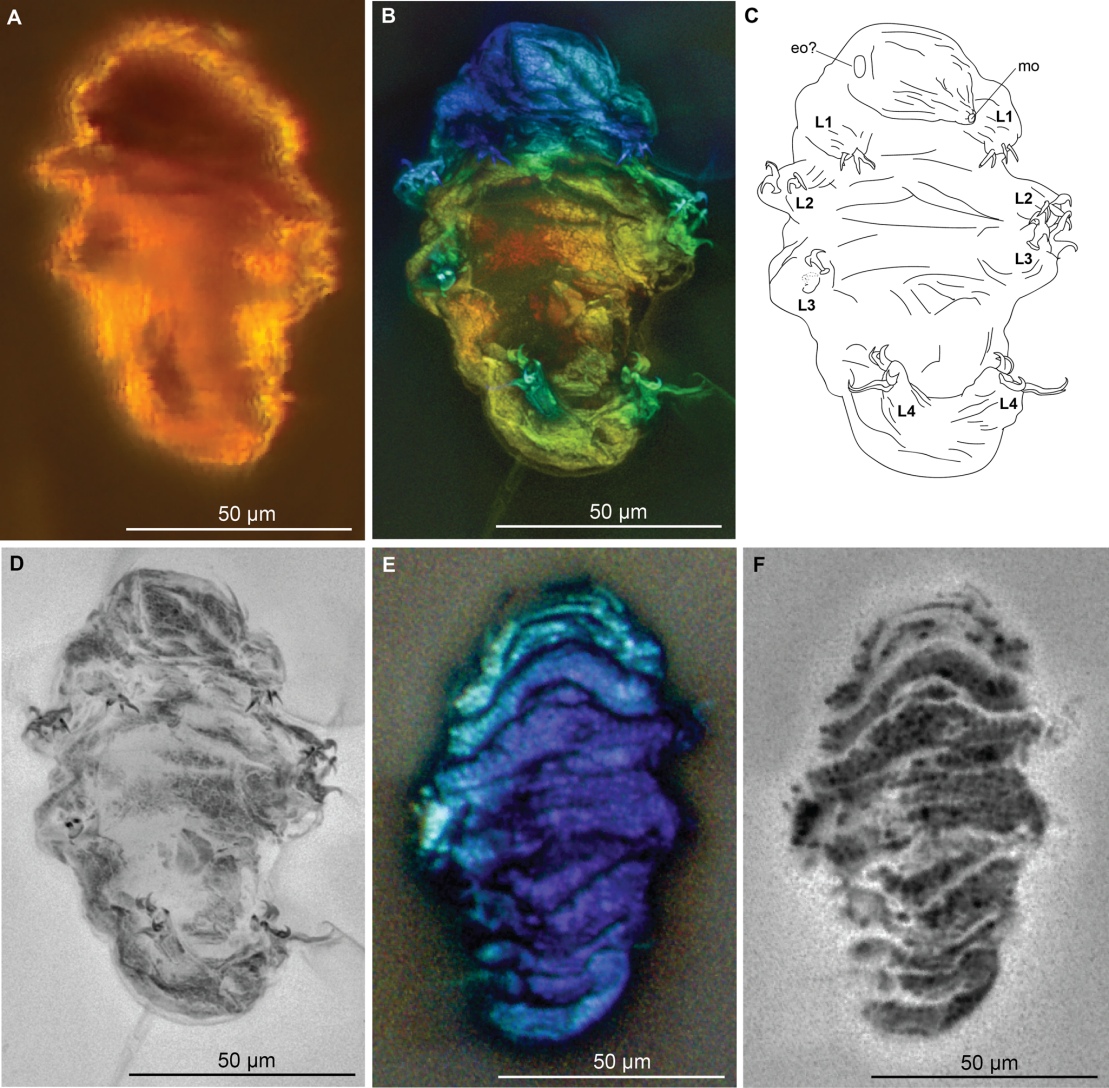
Habitus of *Aerobius dactylus* gen. et sp. nov. (MCZ PALE-45862). *Aerobius dactylus* gen. et sp. nov. (MCZ PALE-45862) in ventral (**A**, **D**) and dorsal view (**E**, **F**). **A** Specimen photographed with transmitted light under compound microscope. **B**, **E** Specimen photographed with autofluorescence under confocal microscope at 639 nm; different colors indicate *z*-depth, with violet to red gradient representing the shallowest to deepest planes, respectively. **C** Schematic drawing. **D**, **F** Specimen and claws viewed in inverted greyscale to highlight autofluorescence intensity (darker—more intense, lighter—least intense). eo elliptical organ, L*n* leg number, mo mouth.

The holotype and only known specimen (MCZ PALE-45862) is a complete body fossil, clearly observable in the dorso-ventral view and appears to be curled-up and shriveled (Fig. 3). At this configuration, the body length is ~100 μm. The cuticle appears to be smooth with no observable protuberances. Cuticular folds are observed on the dorsal side, mostly likely due to its preservation in a shriveled state (Fig. 3E, F). Eyespots were not observed. A faint oval-shaped outline can be observed on the dorso-lateral side of the head region (Figs. 3C, S5). Its distinct outline compared to other parts of the body is more evident in inverted greyscale images (Figs. 3D, S5B) The mouth opening can be observed and appears to be smooth and devoid of peribuccal structures (e.g., peribuccal lamellae and peribuccal papulae, Figs. 3C, S5). It was not possible to visualize the buccal apparatus.

**Fig. 4:**
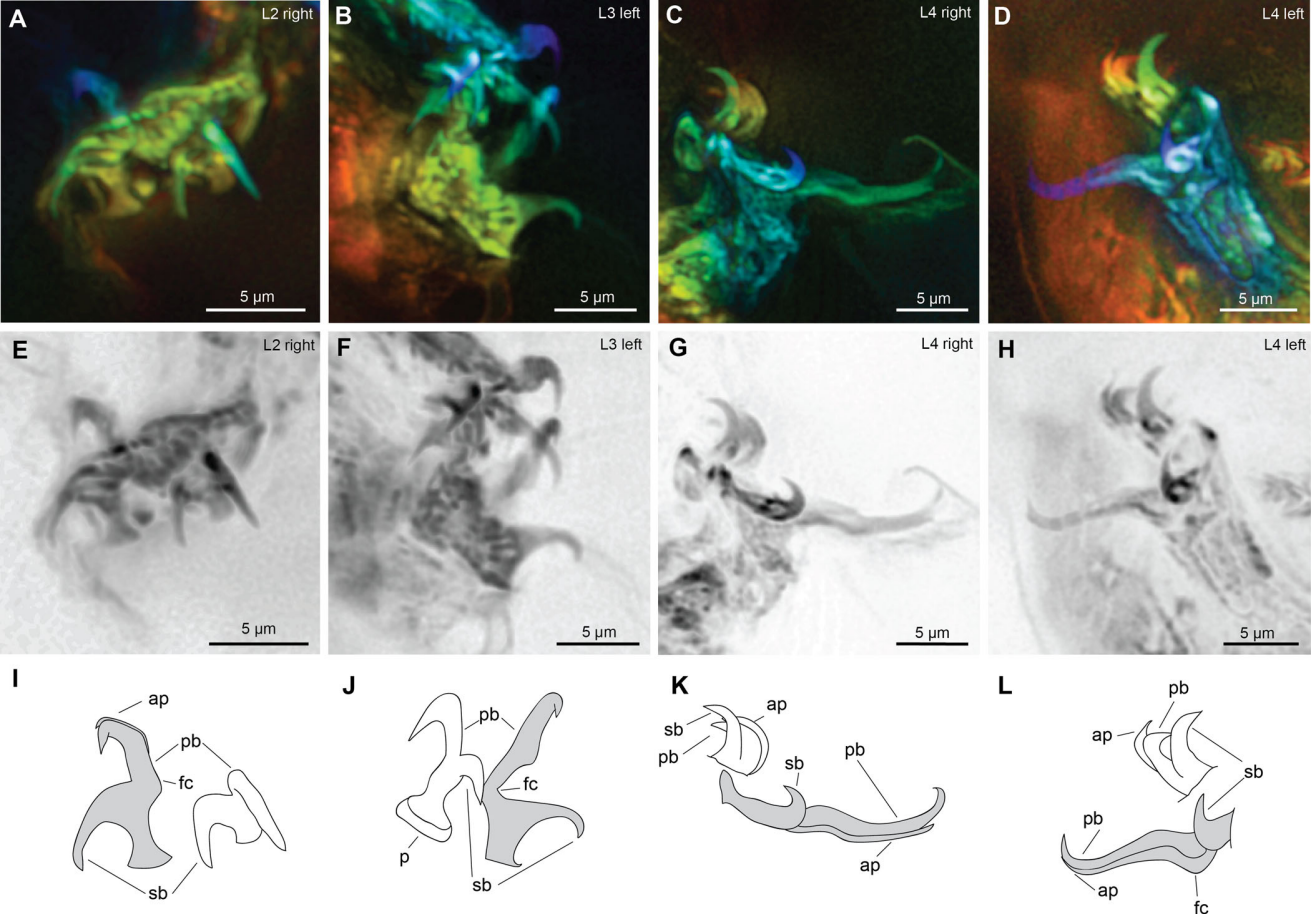
Claws of *Aerobius dactylus* gen. et sp. nov (MCZ PALE-45862) in ventral view. **A**–**D** Structures photographed with autofluorescence under confocal microscope at 639 nm; different colors indicate *z*-depth, with violet to red gradient representing the shallowest to deepest planes, respectively; arrow indicates cuticular extension. **E–H** Claws viewed in inverted greyscale to highlight autofluorescence intensity (darker—more intense, lighter—least intense). **I**–**L** Schematic drawing. Light gray shade—external (legs II and III) and posterior claws (leg IV); unshaded—internal (legs II and III) and anterior claws (leg IV). ap accessory points, fc flexible connection, L*n* leg number, p psedolunule, pb primary branch, sb secondary branch.

Claws can be observed on all legs of MCZ PALE-45862 (Figs. 4, S4). On the first to third legs, the external and internal claws have slightly similar sizes, but their shape differs greatly. The external claws have a modified *Isohypsibius*-type configuration (most evident in claw II) wherein the secondary branch and basal section form a right angle^17^, but the primary branch is connected to the basal section with an evident flexible part, characterized by a curved base of the primary branch (Fig. 4A, B, E, F, I, J), similar to what is observed in *Beo. leggi* (Fig. 2) and other extant tardigrades with *Hypsibius*-type and *Ramazzottius*-type claws (Fig S6). In contrast, the fourth leg pair possess posterior and anterior claws that are greatly different in shape and size. The fourth leg posterior claws appear to be either a typical *Hypsibius*-type claws wherein the secondary branch forms a continuous curve with the basal section or a modified *Isohypsibius*-type claws similar to the first three legs. Unfortunately, the orientation of the claws does not allow us to confidently discern between these two character options. An evident flexible connection between the primary branch and basal section is present (Fig. 4C, D, G, H, K, L). Furthermore, the primary branch of the posterior claw in the fourth leg is notably longer than its associated secondary branch. These differences between the two branches are not obvious in the external claws of the first to third legs. The internal and anterior claws appear to be robust and rigid (Fig. 4). We were not able to obtain reliable measurements of all claws since they were not fully extended (Table S5) due to the preservation of the fossil. Pseudolunules are at least observed in the internal claw (Fig. 4J), while accessory points are observed in the external and posterior claws (Fig. 4I, K, L).

### Phylogenetic affinities and classification of Cretaceous Canadian tardigrade fossils

**Fig. 5:**
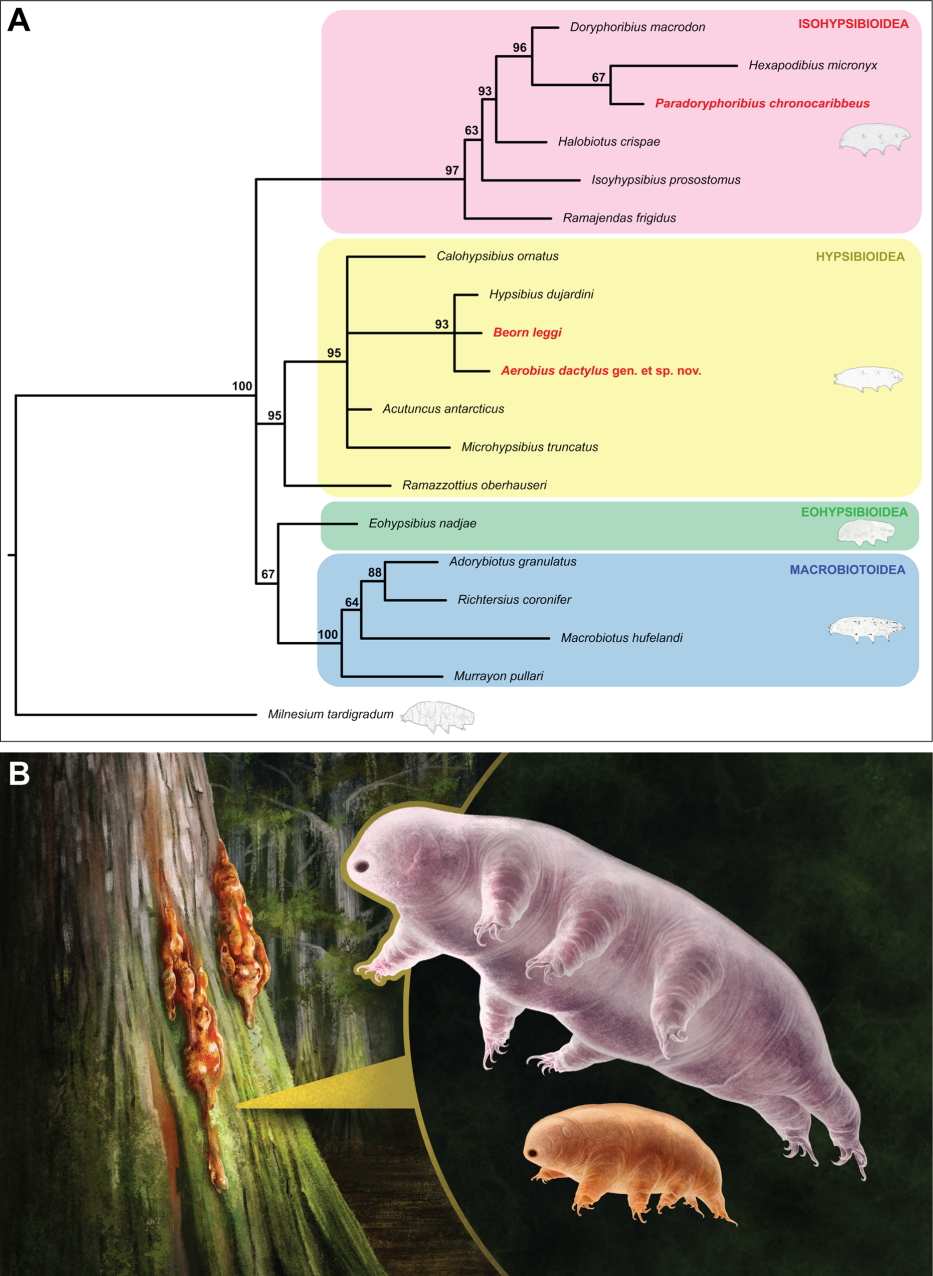
Phylogenetic relationships of *Beorn leggi* and *Aerobius dactylus* gen. et sp. nov. **A** Phylogenetic result of the total evidence approach using 36 morphological characters and 1774 bp 18S rRNA sequences. Numbers at nodes represent posterior probability supports. Fossils are highlighted in red bold texts. **B** Artistic reconstruction of the two fossil specimens by Franz Anthony.

Our total evidence phylogenetic analysis recovered *Beo. leggi* and *Aerobius dactylus* (*Aer. dactylus*) gen. et sp. nov. within the superfamily Hypsibioidea (Fig. 5A). Given the strong relationship of the two fossils to other hypsibioids (e.g., *Hypsibius dujardini*—*Hys. dujardini*) and the presence of *Hypsibius*-type claws, we formally classify them within the superfamily Hypsibioidea (see Systematic Paleontology in the Supplementary Text).

Extant tardigrades with *Hypsibius-*type external claws are exclusively found within the family Hypsibiidae^19^; thus, we reject the extinct family Beornidae^5^, and instead formally reallocate *Beo. leggi* to family Hypsibiidae. The lack of informative buccal apparatus characters in *Beo. leggi* does not allow us to place it within any subfamilies of Hypsibiidae. The downgrading of Beornidae to a subfamily status is also not possible due to the absence of clear synapomorphies. Although the external morphology of *Beo. leggi* is indistinguishable from other hypsibiids, we maintain this taxon because it is not possible to accommodate it within an extant genus. Although a recent study suggested a polyphyletic relationship of the subfamilies of Hypsibiidae^20^, our phylogeny (Figs. S7, S8) recovered a monophyletic Hypsibiidae congruent with most previous results^21–23^. Furthermore, Tumanov & Tsvetkova^20^ suggested elevating subfamilies Itaquasconinae and Pilatobiinae into two separate families, but problematically did not provide a morphological diagnosis. This new proposal was also not recognized by a recent study that redescribed several hypsibiids, including those belonging to subfamilies Itaquasconinae and Pilatobiinae^24^. Hence, we maintain the previous taxonomic rankings of Hypsibiidae *sensu*^21^ (i.e., consisting of four subfamilies) and used this definition for defining the clade to be calibrated in the divergence time estimates.

Our results showed *Aer. dactylus* gen. et sp. nov. in a polytomy with *Beo. leggi* and the hypsibiid *Hys. dujardini* (Fig. 5A). However, we opt to not place it in any extant hypsibioid families due to its different claw morphology. The claw pairs of the hind legs are also notably different in shape and size compared to the rest of the anterior claw pairs. Therefore, we place *Beo. leggi* within the family Hypsibiidae, while *Aer. dactylus* gen. et sp. nov. is placed in an uncertain position outside this family but still within superfamily Hypsibioidea.

Aside from hypsibiids, tardigrades with external claws featuring evident flexible connections (i.e., curved base of the primary branch) are also found in the family Ramazzottiidae^18^ and Ramajendidae^25^. The claws of ramazzottiids (i.e., *Ramazzottius*-type) have a long and slender primary branch and a basal section longer than the secondary branch^17^ (Fig. S6). Since these are not observed in the two fossil specimens, we consider that their inclusion within Ramazzottidae is unlikely. The claws of ramajendids are referred as *Hypsibius*-type^17,25^, but they differ from typical *Hypsibius-*type claws in having a long and slender primary branch similar to *Ramazzottius-*type claws (e.g., Fig. 3 in ref. ^25^). Due to this claw difference and ramajendids being part of superfamily Isohypsibioidea^25^, compared to the fossils clustering within superfamily Hypsibioidea (Fig. 5), we consider the inclusion of the two fossil specimens within Ramajendidae to be unlikely and not well supported by our data.

### Divergence time estimation using *Beorn leggi*

With the newly proposed taxonomic placements of the Cretaceous fossils, we explored the implications of using them for estimating the divergence times of major tardigrade groups. Since *Beo. leggi* is placed at a lower taxonomic group compared to *Aer. dactylus* gen. et sp. nov., we only used this fossil as a calibration point to calibrate the superfamily Hypsibioidea or family Hypsibiidae, depending on the calibration strategy employed.

**Fig. 6:**
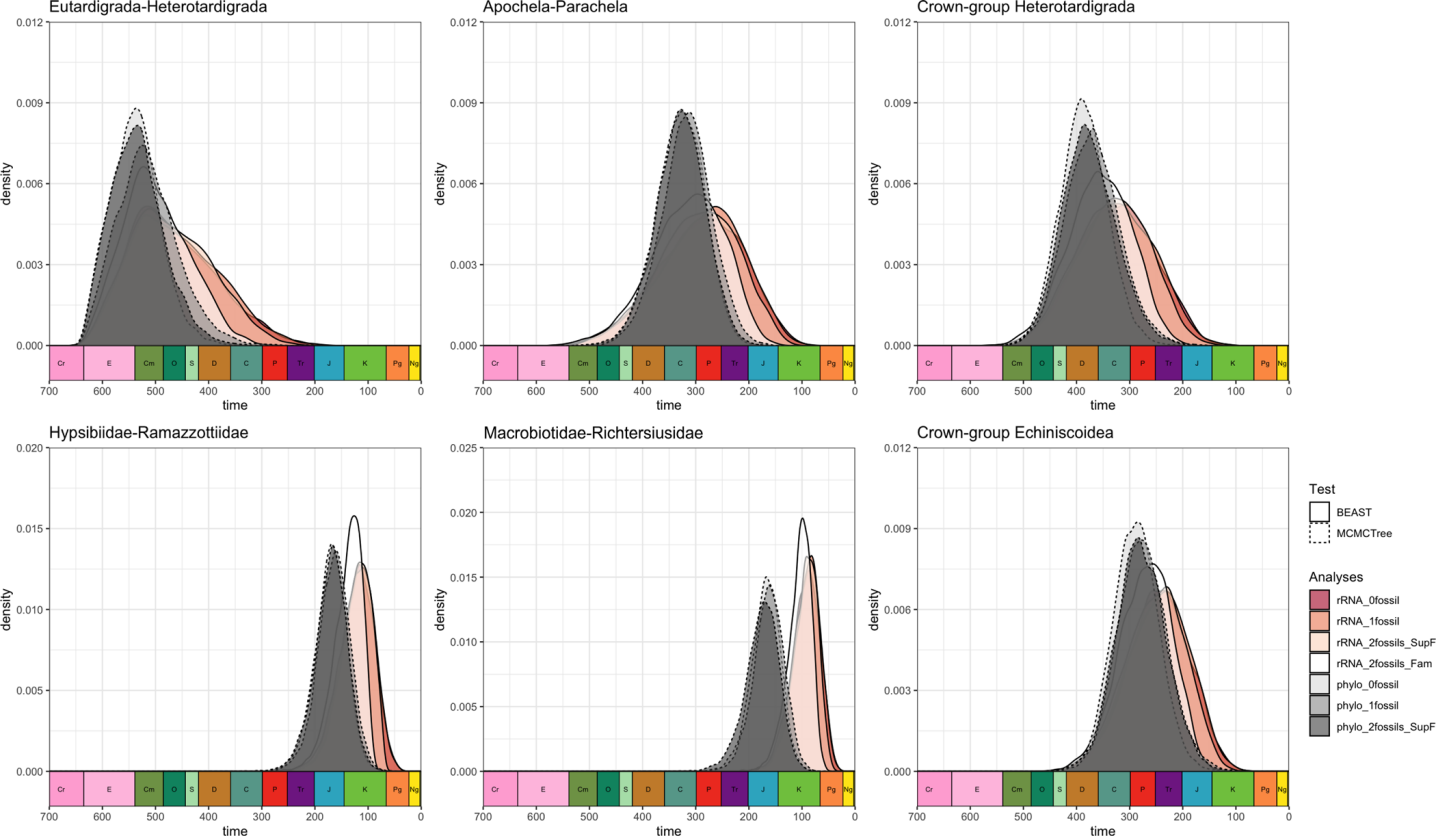
Density plots of posterior divergence time estimates of different tardigrade clades. The upper row represents deeper nodes at the phylum and class levels, while the lower row represents shallower nodes at the order and family levels. Different colors represent different calibration strategies (0fossil—1st strategy, 1fossil—2nd strategy, 2fossils_SupF—3rd strategy, 2fossils_Fam—4th strategy) and dataset used (rRNA—18S/28S rRNA, phylo—phylogenomic). Line type represents the type of analyses used (i.e., BEAST or MCMCtree).

**Fig. 7:**
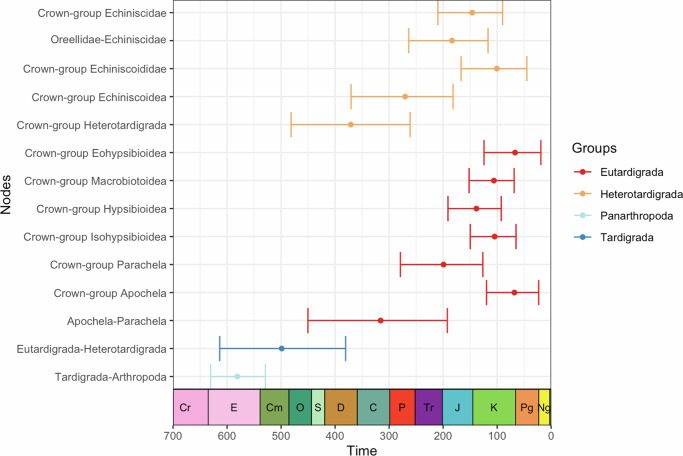
Posterior divergence time estimates obtained using BEAST and the 18S/28S rRNA dataset with *Beorn leggi* used to calibrate at the family level. Dot represents the mean common ancestor value while the error bars mark the minimum and maximum boundary of the 95% height posterior density. Different colors represent major tardigrade taxonomic groups.

Our analyses show that the peaks of the density plots are overlapping at the deepest split of the crown-group tardigrades (i.e., Eutardigrada–Heterotardigrada) (Fig. 6A). At shallower nodes, starting with the split of each of the two classes (i.e., Apochela-Parachela and crown-group Heterotardigrada), the effect of using different datasets is observed (Fig. 6B, C). Analyses using the transcriptome data run in MCMCTree appear to have older estimates compared to those run in BEAST using the 18S/28S ribosomal RNA (rRNA) sequences. The posterior density plots overlap for analyses of both sequence datasets. However, this is not true for one BEAST analysis which corresponds to the strategy that used *Beo. leggi* to calibrate the family Hypsibiidae (4th calibration strategy: 2fossils_Fam). At shallower nodes, this analysis consistently has an older time estimate compared to other BEAST analyses (Figs. 6D–F, S9, S10). Given the different pattern observed using the 4th calibration strategy in BEAST, only the time estimates obtained in this analysis (Figs. 7, S13) are listed below and used for the following discussion.

The split of the Eutardigrada and Heterotardigrada (i.e., crown-group tardigrades) is estimated to occur around the middle Cambrian [mean: 498.86 Mya, 95% HPD: 613.66–380.5 Mya]. The split of the crown-group heterotardigrades is estimated to be earlier [mean: 370.94 Mya, 95% HPD: 481.47–261.14 Mya] compared to the split of the limnoterrestrial eutardigrades (i.e., Apochela-Parachela) [mean: 315.69 Mya, 95% HPD: 450.25–192.23 Mya]. These results correspond to likely splits of heterotardigrades and eutardigrades during the Upper Devonian and Lower Pennsylvanian, respectively. The split of limnoterrestrial heterotardigrades (i.e., oreellids and echiniscids) from the rest of the marine echiniscoidids (i.e., crown-group Echiniscoidea) is estimated to occur around the middle Permian [mean: 270.26 Mya, 95% HPD: 370.39–181.56 Mya]. Except for the crown-group heterotardigrades, these geological time periods correspond to the highest relative frequencies of clade age estimates (Fig. S14A–C, E). Our statistical test also showed a significant difference between the data-inclusive and prior-only frequencies (Table S6).

After the Paleozoic, we observed a pattern wherein the limnoterrestrial echiniscoideans (i.e., Oreellidae-Echiniscidae) and crown-group parachelans, the most speciose group in their respective classes^26–28^, are estimated to split almost at the same time, around the Lower Jurassic [mean: 183.44 Mya, 95% HPD: 263.64–116.83 Mya; and mean: 199.2 Mya, 95% HPD: 279.08–126.49 Mya, respectively]. The same geological period has the highest relative frequencies of age estimates (Fig. S14D, F).

Most of the parachelan superfamilies are estimated to have diverged in the Lower Cretaceous [Hypsibioidea: 138.41 Mya, 95% HPD: 190.99–92.38 Mya; Isohypsibioidea: 104.61 Mya, 95% HPD: 149.74–65.06 Mya; Macrobiotoidea: 106.02 Mya, 95% HPD: 151.89–68.52 Mya]. Only eohypsibioids are estimated to split in the Upper Cretaceous [mean: 66.83 Mya, 95% HPD: 124.27–19.01 Mya]. Crown-group Apochela are also estimated to split at this period [mean: 67.98 Mya, 95% HPD: 119.64–23.06]. Lastly, the split of the limnoterrestrial heterotardigrade family Echiniscidae is estimated to occur around the Upper Jurassic [mean: 146.01, 95% HPD: 209.59–89.93 Mya].

## Discussion

Our new data on Cretaceous tardigrades from Canadian amber reveal new insights about the macroevolution of this group in deep time and allow making further comparisons with other extinct representatives. For instance, in the initial description of *Mil. swolenskyi* the authors emphasized the remarkable degree of morphological stasis in this taxon relative to modern forms and predicted that stratigraphically younger tardigrade fossils would closely resemble extant species^6^. We find some evidence for external morphological stasis in *Beo. leggi* based on the claw structure similar to extant hypsibiids (Fig. 2). However, the discovery of *Aer. dactylus* gen. et sp. nov. could challenge this prediction. If the claws on the hind legs of *Aer. dactylus* gen. et sp. nov. are of the *Hypsibius*-type, it would appear to have a unique combination of claw organization not observed in extant tardigrades. Likewise, the recent description of the even younger *Par. chronocaribbeus* (Miocene: ~16 Mya) also demonstrated a unique morphology in which the buccal apparatus is different from other isohypsibioids^7^, and suggests a high degree of foregut homoplasy among eutardigrades^29^. Taken together, these findings indicate that tardigrade fossils indeed capture macroevolutionary changes, although the overall lobopodous body maintains a high degree of stability as also observed among extant representatives.

The claws of *Aer. dactylus* gen. et sp. nov. offer new insights into the macroevolution of eutardigrades. For example, the modified *Isohypsibius*-type claws show an intermediate morphology between the typical *Isohypisibius*-type and *Hypsibius*-type claws which could reflect an evolutionary transition between these two claw types. If the claws on the hind legs of *Aer. dactylus* gen. et sp. nov are of the *Hypsibius*-type, the claw morphology of *Aer. dactylus* gen. et sp. nov. will appear to be different between the first three pairs of legs and the last pair of legs (Fig. 4). This is a similar pattern observed in extant isohypsibioids such as *Hexapodibius* and *Weglarskobius*^30,31^, and macrobiotoids, such as *Calcarobiotus* (*Discrepunguis*) and *Pseudohexapodibius*^32,33^. These observations suggest that the fourth leg pair can have a different evolutionary history, expressed in both extant and extinct species. This is further supported by different expression patterns of some genes between the first three anterior and posterior limb pairs during embryogenesis^34^. These differences seem to be also expressed in terms of the leg function in eutardigrades, as the first three pairs of legs are used for walking while the last pair is used for grasping into substrates^35,36^, and thus, different claw morphologies could exist between these legs to optimize their functions.

At the deepest split of the tardigrades (Fig. 6A), our results showed that the time estimates were similar, regardless of the type of dataset (i.e., 18S/28S rRNA or transcriptome) or the fossil calibration strategies used. The density plots were also overlapping with the analyses run with the sequences when compared to the prior-only analyses, regardless of the dataset (Figs. S11,S12). This outcome suggests that the age estimations are either influenced by the priors, or that the priors truly reflect the real divergence times. Although it is hard to disentangle these two scenarios, it is worth noting that our Kolmogorov–Smirnov test showed a significant difference, at least with the density plots obtained using BEAST and the fourth calibration (2fossils_Fam), between the data-driven versus prior-only distributions (Table S6). A Cambrian divergence for crown-group tardigrades has been previously estimated using multiple partitioning schemes of a different phylogenomic dataset^2^.

At shallower nodes, specifically starting from family-level splits (Fig. 6D–F), our results indicate that *Beo. leggi* has an effect in estimating divergence time, but only if it is used to calibrate at the family level. This is important since these splits correspond to the divergence of speciose eutardigrade taxa (e.g., *Mesobiotus* and *Paramacrobiotus*)^26–28^ and can help correlate this timing with certain biological events that could explain their abundance. It should be noted that the same family-level calibration was not feasible with phylogenomics due to the lack of samples that allow the calibration at this level (i.e., *Hypsibius exemplaris* is the only available hypsiibid transcriptome). This highlights the need for genomes and transcriptomes of other tardigrades to enable more accurate calibration strategies. Additionally, our results suggest that using outgroup calibrations or doing one-fossil calibration could underestimate divergence times of shallower nodes since these types of analyses in BEAST consistently provided a younger estimate (Fig. S12). This also highlights the importance of redescribing fossils to properly determine their taxonomic positions so they can be accurately used as calibrations. On this note, caution should be done in using *Mil. swolenskyi* as a calibration point for the genus *Milnesium* since images of its internal structures, particularly the buccal apparatus, are lacking to fully ascertain its inclusion in the genus.

With the caveat of overlap with the prior distribution, our results indicate that the divergence of crown-group tardigrades into Heterotardigrada and Eutardigrada could have taken place during the middle Cambrian (Figs. 7, S14). This result implies that the four-legged body plan that defines modern tardigrades most likely evolved during the Cambrian, although it is uncertain whether the group had already achieved its fully miniaturized body size at the time. The absence of crown-group tardigrades in the Cambrian complicates reconstructing the exact body size of early representatives, although it has been suggested that modern tardigrades became miniaturized from a macroscopic ancestor^37^. This is further supported by morphology-based phylogenetic analyses that showed the relationship of macroscopic Cambrian lobopodians (e.g., *Aysheaia pedunculata, Onychodictyon ferox*, and luolishaniids) to the smaller-sized extant tardigrades^38–40^. Although it is difficult to establish a causal explanation, tardigrade miniaturization could have occurred during the Cambrian as a consequence of the rapid ecological diversification of different animal groups and the substrate revolution that significantly affected the composition of benthic communities^41^. In this context, a microscopic body size could be an advantageous strategy to avoid predators and occupy newly oxygenated interstitial zones. Indeed in some animals, predation is cited as a viability cost for having large body sizes^42^. The hypothesized timing also supports the hypothesis that the crown-group tardigrade ancestor was marine in origin^43^.

The two major groups of extant limnoterrestrial tardigrades comprise the eutardigrades and echiniscoidean heterotardigrades, specifically the oreellids and echiniscids. Given that these groups include representatives of the two main branches of the tardigrade tree (Fig. S13^21,44^) this phylogenetic distribution implies that tardigrades underwent at least two independent events of terrestrial colonization. These species, however, still require to be surrounded by liquid water in order to be active^1^. The crown-group eutardigrade clades Apochela and Parachela are estimated to have diverged around the Upper Pennsylvanian of the Carboniferous (Figs. 7, S14). Common limnoterrestrial tardigrade habitats, such as lichens, liverworts, and mosses^4^, were already well established by this period^45^. Indeed, the oldest fossilized lichens and liverworts are dated to the Devonian^46,47^, while diverse groups of ferns were present in the Carboniferous^48^. The presence of these diverse habitats would influence the eutardigrade diversification due to the abundance of substrates to thrive. It should be noted that we infer a long gap between the split of crown-group tardigrades (~499 Mya) and crown-group eutardigrades (~316 Mya) (Fig. S13), which represents the unknown history of this group. Understanding what happened between this time and what tardigrade lineages fill this gap will require additional paleontological discoveries and future investigations.

The divergence of the limnoterrestrial echiniscoideans (i.e., oreellids and echiniscids) is estimated to have occurred much later, specifically around the Lower Jurassic, which also coincides with the split of crown-group parachelans (Figs. 7, S14). These groups correspond to the most speciose clade in their respective classes^26–28^. Our results also showed that echiniscids and parachelan superfamilies diverged around the Jurassic and Cretaceous, respectively (Fig. 7). Interestingly, these ages correspond to estimates of increased diversification rates of liverworts and mosses in mid-Jurassic and mid-Cretaceous, respectively^49^. Macrolichen forms (e.g., foliose), the type of lichens that harbor more tardigrade in the present day compared to crustose microlichens^4^, of Lecanoromycetes are estimated to first appear around the Jurassic-Cretaceous boundary^50^. These bursts of diversification would substantially increase the number of substrates available for limnoterrestrial tardigades, which could have influenced their diversification. Lastly, these ages also support the appearance of modern-looking tardigrade fossils in the Cretaceous and thus, fossils dated around this period are good resources for finding crown-group tardigrade fossils.

Both eutardigrades and echiniscoideans are known to undergo cryptobiosis, a reversible state when metabolic processes come to almost a standstill^51^ in the presence of unfavorable conditions which allow them to survive in extreme environments^3^. Comparative genomic and transcriptomics studies have shown that the echiniscoideans and eutardigrades possess different sets of genes and proteins that are involved in cryptobiosis, suggesting that this protective mechanism evolved independently in tardigrades^52,53^. Indeed, marine non-echiniscoidean heterotardigrades are rarely cryptobionts^3^ with the exception of at least one species^54^. Thus, this ability, at the latest, could have been acquired between Upper Devonian to Lower Jurassic in crown-group echiniscoideans and between Upper Ordovician to Lower Jurassic in eutardigrades, around the estimated divergence of these groups (Figs. 7, S13). Despite the long confidence intervals at these splits, it is worth noting that these intervals encompass ecologically severe extinction events^55^. Thus, the acquisition of cryptobiotic abilities of these tardigrades around this time could be one of the factors that have helped them evade extinction.

Our results show that the Canadian fossils are critical for understanding tardigrade evolution. *Beo. leggi* shows external morphological stasis, similar to *Mil. swolenskyi*, which allows us to place it in the extant eutardigrade family, Hypsibiidae. Molecular dating using *Beo. leggi* allow us to estimate divergence times and hypothesize about the diversification of major tardigrade groups. *Aer. dactylus* gen. et sp. nov., on the other hand, shows a different set of morphological characters from *Beo. leggi* that allowed its formalization as a new taxon. Overall, our study highlights the importance of resolving the taxonomic relationships of these crown-group fossils. Finding more tardigrade fossils will enable the reconstruction of more accurate timelines that will open the clade for comparative analyses. By doing so, we will be able to understand the evolution of tardigrade characters, such as inferring when their cryptobiotic ability evolved and estimating their molecular and morphological rates of change over time.

## Materials and methods

### Studied material and provenance

The studied amber material was part of secondary deposits collected by William M. Legg in 1940 along beaches near the entrance of the Saskatchewan River into Cedar Lake, Manitoba (see ref. ^5^). The fossils are housed at the Entomology Collection at the Museum of Comparative Zoology (MCZ), Harvard University (MCZ PALE-5213 and PALE-45862).

### Microscopy and imaging

The studied fossils were photographed with transmitted light and confocal fluorescence microscopy. The amber specimen was mounted to a slide with dental wax and prepared by putting glycerin (Immersol G, Zeiss) on both sides of the field of view. For transmitted light microscopy, the material was imaged using an Axioscope 5 compound microscope (Zeiss) with Axiocam 208 color camera (Zeiss). Different optical sections were obtained to create the final image and the “Sum Slices” Z-Projection type was used for image reconstructions. For the fluorescence microscopy, autofluorescence of the cuticular structures was detected at an excitation wavelength of 639 nm using the LSM 980 Confocal Microscope with Airyscan 2 detector (Zeiss). Color-coded projections of the optical sections were generated using Fiji 2.0 with the “physics” LUT color scheme. Inverted grayscale projections were also generated to highlight autofluorescence signals. The “Max Intensity” Z-Projection type was used for both image reconstructions. The lighting properties of the images were adjusted using Adobe Lightroom Classic 12.3.

Slides of extant tardigrades were imaged using an Axioscope 5 compound microscope (Zeiss) with Axiocam 208 color camera (Zeiss). Different optical sections were obtained, and the “auto-blend” function of Adobe Photoshop 23.5 was used to create the final image. Figures were assembled using Adobe Illustrator 26.5.

### Morphometric measurements

Body length was measured from the most anterior tip of the body to the most caudal part (excluding the hind legs). Claws were measured according to Beasley et al. ^56^ to obtain the lengths of the primary claw branch, secondary claw branch, and basal section. The *br* ratio or the ratio of the secondary claw branch length to the primary claw branch length was also measured^57^. Morphological features were measured using FIJI 2.0, with all measurements given in micrometers.

### Total evidence phylogenetic analysis

We performed phylogenetic analyses using a total evidence approach to test the placement of the Cretaceous fossils relative to extant eutardigrade superfamilies. We used a modified version of the phenotypic character matrix from Mapalo et al. ^7^, consisting of 36 morphological characters that can be grouped into four sets: body surface, claws, bucco-pharyngeal apparatus, and egg morphology (Data S1; http://morphobank.org/permalink/?P4855). Sequences of the 18S rRNA were used for the molecular dataset (Table S1). Morphological character coding was based on the type species of the genera used, except for *Doryphoribius* and *Ramajendas* due to the lack of 18S rRNA sequences of their type species (Data S2). For the molecular dataset, the 18S rRNA sequences were aligned using MAFFT 7.4^58^ using the L-INS-i algorithm. The alignment was then visualized, and both ends were manually trimmed using Aliview 1.28^59^ which resulted in a final length of 1774 nucleotides (Data S3). Both datasets were then concatenated using Seaview 5.0^60^.

The data matrix (1810 characters total, including 36 morphological and 1774 molecular) was subjected to a Bayesian analysis using MrBayes 3.2^61^. For the morphological set, the Mk model^62^ + Gamma with the coding set to “variable”, which excluded two invariant characters was used. For the molecular set, the GTR model + Gamma + proportion of invariable site (nst = 6, rates = invgamma) was used, based on the best model scheme obtained using Partitionfinder 2.1^63^ under the Akaike information criterion. The analysis was run for 2,000,000 generations sampling every 500 generations and with a 25% burn-in frequency. Two runs were simultaneously done with each having one cold and three heated chains. Convergence was assessed by checking that the average deviation of split frequencies of the two runs were <0.01, effective sample size values were >200 and the potential scale reduction factor was approximately = 1. A 50% majority rule consensus tree was then obtained to summarize the resulting analysis.

### Divergence time estimation

We tested the implications of the new fossil data as calibration points for estimating the divergence of crown-group Tardigrada. The analyses used different combinations of two datasets—phylogenomic and 18S/28S rRNA barcodes with different sampling sizes. For each analysis, three different fossil node calibration strategies were used: (1) no tardigrade fossils were used with time estimation relying only on the root calibration (0fossil), (2) using *Mil. swolenskyi* as the sole calibration point for the entire crown-group of tardigrades (1fossil), and (3) using *Mil. swolenskyi* as a calibration point for the more precise crown-group eutardigrades and *Beo. leggi* for the clade corresponding to the superfamily Hypsibioidea (2fossils_SupF). An additional strategy was done for the 18S/28S rRNA dataset similar to the third strategy but using *Beo. leggi* as a calibration point for the clade corresponding to the family Hypsibiidae (within Hypsibioidea) (2fossils_Fam). Complete details about the list of species included in the calibrated clades and calibration ages are in Tables S2 and S3.

For the phylogenomic dataset, translated gene sequences from nine tardigrades representing all four major tardigrade groups and one euarthropod (*Drosophila melanogaster*—*D. melanogaster*) as an outgroup were used. Gene homology searches between all the transcriptomes were done using OMA 2.1^64^. After selecting genes that have at least 90% taxa occupancy, 335 orthologs were obtained, aligned, and concatenated. This resulted in a matrix with a length of 139,117 amino acid sites (Data S4). To determine the tardigrade topology, gene trees were first obtained from each of the 335 aligned gene homologs using IQTree 1.6^65^. All the gene trees were then concatenated, and the resulting matrix was used as an input for ASTRAL 4.10^66^. Divergence time estimates were calculated using the approximate likelihood method in MCMCTree^67^ in the PAML 4.9 package^68^ using the independent clock rate model with Birth–Death (BD) tree model. Node calibration was done using uniform distribution for the age priors. Two runs were done for each fossil calibration strategy and convergence was assessed by plotting the time estimates from the two runs and was confirmed if their *R*^2^ value was ~1 (Data S5). Since both runs showed comparable values, only the values from one run were used and shown in the succeeding results.

For the 18S/28S rRNA dataset, 139 tardigrades representing all tardigrade orders were used. When possible, each genus is represented by two species and the samples selected must have at least an 18S rRNA sequence. For the 28S rRNA, only overlapping sequences corresponding to one region of 28 S was used. As a result, 139 sequences of 18S rRNA and 80 sequences of 28S rRNA were used (Data S6). One euarthropod (*D. melanogaster*) was used as an outgroup for all analyses. After each rRNA sequences were individually aligned and trimmed, they were concatenated and resulted in a dataset with a length of 3210 nucleotides (Data S7). The tree topology was reconstructed using maximum likelihood (ML) and Bayesian inference (BI) using the best model scheme obtained from Partitionfinder 2.1. The ML tree was reconstructed using IQTree 1.6 with the matrix divided into two partitions corresponding to each rRNA sequence, and the GTR + I + G model was used for each partition. Bootstrap analysis was done using 1000 replicates. The BI tree was reconstructed using MrBayes 3.2. The matrix was partitioned according to the different rRNA sequences, and the GTR model + Gamma + proportion of invariable site for each partition was used. Convergence was assessed and consensus tree was obtained as for total evidence analysis. Divergence time estimation was done using BEAST 2.6^69^ with the relaxed log normal clock model and BD tree model. The dataset was partitioned based on the type of rRNA sequences and BmodelTest^70^ was used to select the substitution model for each partition. Node calibration was done using uniform distribution for the age priors. For each fossil calibration strategy, three individual runs were done. To make the calculation of the summary tree faster, the log and tree files from all three runs were combined, resampled at every 50,000 generations, and cleansed off the first 25% burn-in values using LogCombiner (for a total of 4503 estimates). Convergence was assessed by checking the log files in Tracer 1.7^71^ and was confirmed if the ESS values are >200 for all statistics. Using the newly resampled tree file, TreeAnnotator from the BEAST package was used to obtain a maximum clade credibility using Common Ancestor (CA) heights as the node heights to produce the final tree containing the divergence time estimates. For each calibration strategy, three runs of exclusive sampling from the priors were performed.

To compare the divergence time estimates obtained between the analyses using the two datasets, density plots were made using R. Detailed methods for the phylogenetic analysis are in the Supplementary Text.

We further dissected density plots of the fourth calibration strategy (2fossil_Fam) with wide ranges by determining the different age estimates of a clade in the posterior tree samples (i.e., a total of 4503 trees from the final “.trees” file) and calculating the relative frequency of their corresponding geological time periods. This allowed us to visualize how often the time of clade divergence is estimated to be within a geological time period across the sampled posterior age estimates. A Kolmogorov–Smirnov test was done to determine if the posterior and prior distributions were statistically different.

### Reporting summary

Further information on research design is available in the Nature Portfolio Reporting Summary linked to this article.

## Supplementary information

Supplementary Information

Description of additional supplementary files

Data S1: Morphological characters used for the total - evidence phylogenetic analysis

Data S2: Character matrix used for the total - evidence phylogenetic analysis

Data S3: 18S alignment used for the total - evidence phylogenetic analysis

Data S4: Translated transcri ptome alignment used for MCMCtree analaysis

Data S5: Results of the MCMCTree runs and convergence test

Data S6: 18S and 28S rRNA Genbank Accession numbers used for the divergence time estimation analyses

Data S7: Concatenated 18S and 28S rRNA sequences used for the divergence time estimation analyses

Reporting Summary

## Data Availability

All data files supporting the findings of this study are available within the published manuscript and the Supplementary Materials (i.e., 18S and 28S rRNA accession numbers, morphological character list, sequence alignments, and MCMCTree runs and convergence tests). All raw files from the phylogenetic analysis (i.e., t, p, and tree files from MrBayes, output tree from ASTRAL and IQTree, mcmc files from MCMCTree, and tre and log files from BEAST), RScripts used, and assembled *Actinarctus doryphorus* genome are available on Dryad Digital Repository (10.5061/dryad.s1rn8pkfx)^72^. Character matrix is also available on MorphoBank (http://morphobank.org/permalink/?P4855). Zoobank registration for *Aerobius dactylus*: http://www.zoobank.org/urn:lsid:zoobank.org:pub:E407CAA2-4928-4670-AB33-F4E5E9E4A589. All data can also be requested from the corresponding authors.

## References

[1] Møbjerg, N., Jørgensen, A., Kristensen, R. M. & Neves, R. C. Morphology and functional anatomy. in *Water Bears: The Biology of Tardigrades* (ed. Schill, R. O.) 57–94 (Springer Nature, Switzerland, 2018).

[2] Howard, R. J. et al. The Ediacaran origin of Ecdysozoa: integrating fossil and phylogenomic data. *J. Geol. Soc.* **179**, jgs2021-107 (2022).

[3] Møbjerg, N. et al. Survival in extreme environments—on the current knowledge of adaptations in tardigrades. *Acta Physiol.* **202**, 409–420 (2011). 10.1111/j.1748-1716.2011.02252.x 21251237

[4] Nelson, D. R., Bartels, P. J. & Guil, N. Tardigrade ecology. in *Water Bears: The Biology of Tardigrades* (ed. Schill, R. O.) 163–210 (Springer Nature, Switzerland, 2018).

[5] Cooper, K. W. The first fossil tardigrade: *Beorn leggi* Cooper, from Cretaceous amber. *Psyche* **71**, 41–48 (1964).

[6] Bertolani, R. & Grimaldi, D. A new eutardigrade (Tardigrada: Milnesiidae) in amber from the Upper Cretaceous (Turonian) of New Jersey. In *Studies on Fossils in Amber, with Particular Reference to the Cretaceous of New Jersey* 103–110 (Backhuys, 2000).

[7] Mapalo, M. A., Robin, N., Boudinot, B. E., Ortega-Hernández, J. & Barden, P. A tardigrade in Dominican amber. *Proc. R. Soc. B Biol. Sci*. **288**, 20211760 (2021). 10.1098/rspb.2021.1760 PMC8493197 34610770

[8] Perry, E., Miller, W. R. & Kaczmarek, Ł. Recommended abbreviations for the names of genera of the phylum Tardigrada. *Zootaxa* **4608**, 145–154 (2019). 10.11646/zootaxa.4608.1.8 31717165

[9] Perry, E., Miller, W. R. & Kaczmarek, Ł. Additional recommended abbreviations for the names of genera of the phylum Tardigrada. *Zootaxa* **4981**, 398–400 (2021). 10.11646/zootaxa.4981.2.12 34186713

[10] Guidetti, R. & Bertolani, R. Paleontology and molecular dating. In *Water Bears: The Biology of Tardigrades* (ed. Schill, R. O.) 131–143 (Springer Nature, Switzerland, 2018).

[11] Regier, J. C., Shultz, J. W., Kambic, R. E. & Nelson, D. R. Robust support for tardigrade clades and their ages from three protein-coding nuclear genes. *Invertebr. Biol.* **123**, 93–100 (2004).

[12] Rota-Stabelli, O., Daley, A. C. & Pisani, D. Molecular timetrees reveal a Cambrian colonization of land and a new scenario for ecdysozoan evolution. *Curr. Biol.* **23**, 392–398 (2013). 23375891 10.1016/j.cub.2013.01.026

[13] Maas, A. & Waloszek, D. Cambrian derivatives of the early arthropod stem lineage, pentastomids, tardigrades and lobopodians—an ‘Orsten’ perspective. *Zool. Anz. J. Comp. Zool.* **240**, 451–459 (2001).

[14] Müller, K. J., Walossek, D. & Zakharov, A. ‘Orsten’ type phosphatized soft-integument preservation and a new record from the Middle Cambrian Kuonamka Formation in Siberia. *Neues Jahrb. Geol. Paläontol. Abh.* **197**, 101–118 (1995).

[15] Guidetti, R., McInnes, S. J., Cesari, M., Rebecchi, L. & Rota-Stabelli, O. Evolutionary scenarios for the origin of an Antarctic tardigrade species based on molecular clock analyses and biogeographic data. *Contrib. Zool.* **86**, 97–110 (2017).

[16] Morek, W., Surmacz, B., López-López, A. & Michalczyk, Ł. “Everything is not everywhere”: time-calibrated phylogeography of the genus *Milnesium* (Tardigrada). *Mol. Ecol.* **30**, 3590–3609 (2021). 33966339 10.1111/mec.15951 PMC8361735

[17] Pilato, G. & Binda, M. G. Definition of families, subfamilies, genera and subgenera of the Eutardigrada and keys to their identification. *Zootaxa* **2404**, 1–54 (2010).

[18] Marley, N. J., McInnes, S. J. & Sands, C. J. Phylum Tardigrada: a re-evaluation of the Parachela. *Zootaxa* **64**, 51–64 (2011).

[19] Degma, P. & Guidetti, R. Tardigrade taxa. in *Water Bears: The Biology of Tardigrades* (ed. Schill, R. O.) 371–409 (Springer Nature, Switzerland, 2018).

[20] Tumanov, D. V. & Tsvetkova, A. Y. Some have drops and some do not, but can we rely on that? Re-investigation of Diphascon tenue (Tardigrada: Eutardigrada) with discussion of the phylogeny and taxonomy of the superfamily Hypsibioidea. *Zoosyst. Ross.* **32**, 50–74 (2023).

[21] Bertolani, R. et al. Phylogeny of Eutardigrada: new molecular data and their morphological support lead to the identification of new evolutionary lineages. *Mol. Phylogenet. Evol.* **76**, 110–126 (2014). 24657804 10.1016/j.ympev.2014.03.006

[22] Tumanov, D. V. Integrative redescription of *Hypsibius pallidoides* Pilato et al., 2011 (Eutardigrada: Hypsibioidea) with the erection of a new genus and discussion on the phylogeny of Hypsibiidae. *Eur. J. Taxon.* **2020**, 1–37 (2020).

[23] Vecchi, M. et al. Expanding Acutuncus: Phylogenetics and morphological analyses reveal a considerably wider distribution for this tardigrade genus. *Mol. Phylogenet. Evol.* **180**, 107707 (2023). 36681365 10.1016/j.ympev.2023.107707

[24] Gąsiorek, P., Blagden, B., Morek, W. & Michalczyk, Ł. What is a ‘strong’ synapomorphy? Redescriptions of Murray’s type species and descriptions of new taxa challenge the systematics of Hypsibiidae (Eutardigrada: Parachela). *Zool. J. Linn. Soc*. **zlad151**, 1–63 (2023).

[25] Tumanov, D. V. End of a mystery: integrative approach reveals the phylogenetic position of an enigmatic Antarctic tardigrade genus *Ramajendas* (Tardigrada, Eutardigrada). *Zool. Scr.* **51**, 217–231 (2022).

[26] Degma, P. & Guidetti, R. Notes to the current checklist of Tardigrada. *Zootaxa* **1579**, 41–53 10.11646/zootaxa.1579.1.2 (2007).

[27] Degma, P. & Guidetti, R. *Actual checklist of Tardigrada species* 1–67 10.25431/11380 (2023).

[28] Guidetti, R. & Bertolani, R. Tardigrade taxonomy: an updated check list of the taxa and a list of characters for their identification. *Zootaxa* **46**, 1–46 (2005).

[29] Guil, N., Machordom, A. & Guidetti, R. High level of phenotypic homoplasy amongst eutardigrades (tardigrada) based on morphological and total evidence phylogenetic analyses. *Zool. J. Linn. Soc.* **169**, 1–26 (2013).

[30] Pilato, G. Su un interessante Tardigrado esapodo delle dune costiere siciliane: *Hexapodibius micronyx* n. gen. n. sp. *Boll. Accad. Gioenia Sci. Nat. Catania* **9**, 619–622 (1969).

[31] Kaczmarek, Ł., Bartylak, T. & Roszkowska, M. Two new genera of long clawed Isohypsibioidea Guil, Jørgensen & Kristensen, 2019. *Zootaxa* **4729**, 293–299 (2020). 10.11646/zootaxa.4729.2.10 32229867

[32] Bertolani, R. & Biserov, V. I. Leg and claw adaptations in soil tardigrades, with erection of two new genera of Eutardigrada, Macrobiotidae: *Pseudohexapodibius* and *Xerobiotus*. *Invertebr. Biol.* **115**, 299–304 (1996).

[33] Guidetti, R. & Bertolani, R. An evolutionary line of the Macrobiotinae (Tardigrada, Macrobiotidae): *Calcarobiotus* and related species. *Ital. J. Zool.* **68**, 229–233 (2001).

[34] Game, M. & Smith, F. W. Loss of intermediate regions of perpendicular body axes contributed to miniaturization of tardigrades: tardigrade leg patterning. *Proc. R. Soc. B Biol. Sci*. **287**, 20201135 (2020). 10.1098/rspb.2020.1135 PMC7423659 33043863

[35] Schüttler, L. & Greven, H. Beobachtungen zur Lokomotion von Tardigraden. *Acta Biol. Benrodis* **11**, 33–52 (2000).

[36] Nirody, J. A., Duran, L. A., Johnston, D. & Cohen, D. J. Tardigrades exhibit robust interlimb coordination across walking speeds and terrains. *Proc. Natl Acad. Sci. USA* **118**, 1–9 (2021). 10.1073/pnas.2107289118 PMC8536314 34446560

[37] Smith, F. W. et al. The compact body plan of tardigrades evolved by the loss of a large body region. *Curr. Biol.* **26**, 224–229 (2016). 26776737 10.1016/j.cub.2015.11.059

[38] Kihm, J.-H. et al. Cambrian lobopodians shed light on the origin of the tardigrade body plan. *Proc. Natl Acad. Sci. USA* **120**, 2017 (2023). 10.1073/pnas.2211251120 PMC10334802 37399417

[39] Smith, M. R. & Ortega-Hernández, J. *Hallucigenia’s* onychophoran-like claws and the case for Tactopoda. *Nature* **514**, 363–366 (2014). 25132546 10.1038/nature13576

[40] Yang, J. et al. A superarmored lobopodian from the Cambrian of China and early disparity in the evolution of Onychophora. *Proc. Natl Acad. Sci. USA* **112**, 8678–8683 (2015). 26124122 10.1073/pnas.1505596112 PMC4507230

[41] Mángano, M. G. & Buatois, L. A. The Cambrian revolutions: trace-fossil record, timing, links and geobiological impact. *Earth Sci. Rev.* **173**, 96–108 (2017).

[42] Blanckenhorn, W. U. The evolution of body size: what keeps organisms small? *Q. Rev. Biol.* **75**, 385–407 (2000). 11125698 10.1086/393620

[43] Renaud-Mornant, J. Species diversity in marine Tardigrada. In *Third International Symposium on the Tardigrada* 149–178 (East Tennessee State University, 1982).

[44] Guil, N., Jørgensen, A. & Kristensen, R. An upgraded comprehensive multilocus phylogeny of the Tardigrada tree of life. *Zool. Scr.* **48**, 120–137 (2019).

[45] Morris, J. L. et al. The timescale of early land plant evolution. *Proc. Natl Acad. Sci. USA* **115**, E2274–E2283 (2018). 29463716 10.1073/pnas.1719588115 PMC5877938

[46] Hernick, L. V. A., Landing, E. & Bartowski, K. E. Earth’s oldest liverworts-*Metzgeriothallus sharonae* sp. nov. from the Middle Devonian (Givetian) of eastern New York, USA. *Rev. Palaeobot. Palynol.* **148**, 154–162 (2008).

[47] Honegger, R., Edwards, D. & Axe, L. The earliest records of internally stratified cyanobacterial and algal lichens from the Lower Devonian of the Welsh Borderland. *N. Phytol.* **197**, 264–275 (2013). 10.1111/nph.12009 23110612

[48] Dimichele, W. A. & Phillips, T. L. The ecology of Paleozoic ferns. *Rev. Palaeobot. Palynol.* **119**, 143–159 (2002).

[49] Laenen, B. et al. Extant diversity of bryophytes emerged from successive post-Mesozoic diversification bursts. *Nat. Commun.* **5**, 1–6 (2014). 10.1038/ncomms6134 25346115

[50] Nelsen, M. P., Lücking, R., Boyce, C. K., Lumbsch, H. T. & Ree, R. H. The macroevolutionary dynamics of symbiotic and phenotypic diversification in lichens. *Proc. Natl Acad. Sci. USA* **117**, 21495–21503 (2020). 32796103 10.1073/pnas.2001913117 PMC7474681

[51] Keilin, D. The problem of anabiosis or latent life: history and current concept author. *Proc. R. Soc. B Biol. Sci.* **150**, 149–191 (1959). 13633975 10.1098/rspb.1959.0013

[52] Kamilari, M., Jørgensen, A., Schiøtt, M. & Møbjerg, N. Comparative transcriptomics suggest unique molecular adaptations within tardigrade lineages. *BMC Genomics* **20**, 607 (2019). 31340759 10.1186/s12864-019-5912-x PMC6652013

[53] Murai, Y. et al. Multiomics study of a heterotardigrade, *Echiniscus testudo*, suggests the possibility of convergent evolution of abundant heat-soluble proteins in Tardigrada. *BMC Genomics* **22**, 1–14 (2021). 34763673 10.1186/s12864-021-08131-x PMC8582207

[54] Jørgensen, A. & Møbjerg, N. Notes on the cryptobiotic capability of the marine arthrotardigrades *Styraconyx haploceros* (Halechiniscidae) and *Batillipes pennaki* (Batillipedidae) from the tidal zone in Roscoff, France. *Mar. Biol. Res.* **11**, 214–217 (2015).

[55] McGhee, G. R., Clapham, M. E., Sheehan, P. M., Bottjer, D. J. & Droser, M. L. A new ecological-severity ranking of major Phanerozoic biodiversity crises. *Palaeogeogr. Palaeoclimatol. Palaeoecol.* **370**, 260–270 (2013).

[56] Beasley, C. W., Kaczmarek, Ł. & Michalczyk, Ł. Doryphoribius mexicanus, a new species of Tardigrada (Eutardigrada: Hypsibiidae) from Mexico (North America). *Proc. Biol. Soc. Wash.* **121**, 34–40 (2008).

[57] Gąsiorek, P., Stec, D., Morek, W. & Michalczyk, Ł. Deceptive conservatism of claws: distinct phyletic lineages concealed within Isohypsibioidea (Eutardigrada) revealed by molecular and morphological evidence. *Contrib. Zool.* **88**, 78–132 (2019).

[58] Katoh, K. & Standley, D. M. MAFFT multiple sequence alignment software version 7: improvements in performance and usability. *Mol. Biol. Evol.* **30**, 772–780 (2013). 23329690 10.1093/molbev/mst010 PMC3603318

[59] Larsson, A. AliView: a fast and lightweight alignment viewer and editor for large datasets. *Bioinformatics* **30**, 3276–3278 (2014). 25095880 10.1093/bioinformatics/btu531 PMC4221126

[60] Gouy, M., Tannier, E., Comte, N. & Parsons, D. P. Seaview version 5: a multiplatform software for multiple sequence alignment, molecular phylogenetic analyses, and tree reconciliation. In *Multiple Sequence Alignment: Methods and Protocols, Methods in Molecular Biology* Vol. 1656 (ed. Katoh, K.) 241–260 (Humana, 2021). 10.1007/978-1-0716-1036-7_15 33289897

[61] Ronquist, F. et al. MrBayes 3.2: efficient Bayesian phylogenetic inference and model choice across a large model space. *Syst. Biol.* **61**, 539–542 (2012). 22357727 10.1093/sysbio/sys029 PMC3329765

[62] Lewis, P. O. A likelihood approach to estimating phylogeny from discrete morphological character data. *Syst. Biol.* **50**, 913–925 (2001). 12116640 10.1080/106351501753462876

[63] Lanfear, R., Frandsen, P. B., Wright, A. M., Senfeld, T. & Calcott, B. PartitionFinder 2: new methods for selecting partitioned models of evolution for molecular and morphological phylogenetic analyses. *Mol. Biol. Evol.* **34**, 772–773 (2017). 28013191 10.1093/molbev/msw260

[64] Train, C. M., Glover, N. M., Gonnet, G. H., Altenhoff, A. M. & Dessimoz, C. Orthologous Matrix (OMA) algorithm 2.0: more robust to asymmetric evolutionary rates and more scalable hierarchical orthologous group inference. *Bioinformatics* **33**, i75–i82 (2017). 28881964 10.1093/bioinformatics/btx229 PMC5870696

[65] Nguyen, L. T., Schmidt, H. A., Von Haeseler, A. & Minh, B. Q. IQ-TREE: a fast and effective stochastic algorithm for estimating maximum-likelihood phylogenies. *Mol. Biol. Evol.* **32**, 268–274 (2015). 25371430 10.1093/molbev/msu300 PMC4271533

[66] Mirarab, S. & Warnow, T. ASTRAL-II: coalescent-based species tree estimation with many hundreds of taxa and thousands of genes. *Bioinformatics* **31**, i44–i52 (2015). 26072508 10.1093/bioinformatics/btv234 PMC4765870

[67] Reis, M. Dos & Yang, Z. Approximate likelihood calculation on a phylogeny for Bayesian estimation of divergence times. *Mol. Biol. Evol.* **28**, 2161–2172 (2011). 21310946 10.1093/molbev/msr045

[68] Yang, Z. PAML 4: phylogenetic analysis by maximum likelihood. *Mol. Biol. Evol.* **24**, 1586–1591 (2007). 17483113 10.1093/molbev/msm088

[69] Bouckaert, R. R. et al. BEAST 2.5: an advanced software platform for Bayesian evolutionary analysis. *PLoS Comput. Biol.* **15**, 1–28 (2019). 10.1371/journal.pcbi.1006650 PMC6472827 30958812

[70] Bouckaert, R. R. & Drummond, A. J. bModelTest: Bayesian phylogenetic site model averaging and model comparison. *BMC Evol. Biol.* **17**, 1–11 (2017). 28166715 10.1186/s12862-017-0890-6 PMC5294809

[71] Rambaut, A., Drummond, A. J., Xie, D., Baele, G. & Suchard, M. A. Posterior summarization in Bayesian phylogenetics using Tracer 1.7. *Syst. Biol.* **67**, 901–904 (2018). 29718447 10.1093/sysbio/syy032 PMC6101584

[72] Mapalo, M. A., Wolfe, J. M. & Ortega-Hernández, J. Cretaceous amber inclusions illuminate the evolutionary origin of tardigrades. Raw Files. Dryad Digital Repository. 10.5061/dryad.s1rn8pkfx (2024). 10.1038/s42003-024-06643-2 PMC11303527 39107512

