# Author Correction: Cretaceous amber inclusions illuminate the evolutionary origin of tardigrades

**DOI:** 10.1038/s42003-024-06870-7

**Published:** 2024-09-16

**Authors:** Marc A. Mapalo, Joanna M. Wolfe, Javier Ortega-Hernández

**Affiliations:** Museum of Comparative Zoology and Department of Organismic and Evolutionary Biology, Harvard University, Cambridge, MA USA

**Keywords:** Palaeontology, Phylogenetics, Taxonomy

**Correction to:** *Communications Biology* 10.1038/s42003-024-06643-2, published online 6 August 2024

The original version of the article contained three errors in the text of the “Results” and “Discussion” sections.

In the third paragraph under the section “Divergence time estimation using *Beorn leggi*” of the “Results” section, the original version of the article incorrectly stated, “…compared to the split of the limnoterrestrial eutardigrades (i.e., Apochela-Parachela) [mean: 315.69 Mya, 95% HPD: 431.36–191.08 Mya].” The correct values in the text should read, “…compared to the split of the limnoterrestrial eutardigrades (i.e., Apochela-Parachela) [mean: 315.69 Mya, 95% HPD: 450.25–192.23 Mya].”

In the sixth paragraph of the “Discussion” section, the original version of the article incorrectly stated, “It should be noted that we infer a long gap between the split of crown-group tardigrades (~504 Mya) and crown-group eutardigrades (~307 Mya) (Fig. S13), which represents the unknown history of this group”. The correct values in the text should read, “It should be noted that we infer a long gap between the split of crown-group tardigrades (~499 Mya) and crown-group eutardigrades (~316 Mya) (Fig. S13), which represents the unknown history of this group.”

Lastly, in the second to last paragraph of the “Discussion” section, the original version of the article incorrectly stated, “Thus, this ability, at the latest, could have been acquired between Upper Devonian to Lower Jurassic in crown-group echiniscoideans and between Middle Silurian to Lower Jurassic in eutardigrades, around the estimated divergence of these groups (Figs. 7, S13).” The correct text should read, “Thus, this ability, at the latest, could have been acquired between Upper Devonian to Lower Jurassic in crown-group echiniscoideans and between Upper Ordovician to Lower Jurassic in eutardigrades, around the estimated divergence of these groups (Figs. 7, S13).”

This has now been corrected in the PDF and HTML versions of the article.

