# Supplementary material for: Cretaceous amber inclusions illuminate the evolutionary origin of tardigrades
Source: Commun Biol. 2024 Aug 6;7:953. doi: 10.1038/s42003-024-06643-2 (PMC11303527; doi:10.1038/s42003-024-06643-2)
Supplement: Supplementary file 1 — Supplementary Information [file 42003_2024_6643_MOESM1_ESM.pdf]

Supplementary Text

I. Detailed Materials and Methods

A. Total Evidence Phylogenetic Analysis

To test the placement of the fossil relative to extant eutardigrade superfamilies, we performed phylogenetic analyses using a total evidence approach. For the morphological dataset, the character matrix from Mapalo et al., 2021[1] was modified and consisted of 36 morphological characters that can be grouped into four sets: body surface (three characters), claws (21 characters), bucco-pharyngeal apparatus (10 character) and egg morphology (one character) (Data S1), while for the molecular dataset, sequences of the 18S ribosomal RNA (rRNA) were used (Table S1). Because the goal of the analyses was to assess the superfamily affinity of the fossils, only type genera of the extant families within the four eutardigrade superfamilies (Eohypsibioidea, Hypsibioidea, Isohypsibioidea and Macrobiotoidea) with available 18S rRNA sequences were used, together with *Milnesium tardigradum* as an outgroup to Parachela following the molecular-based tree topology in [2,3]. *Paradoryphoribius chronocaribbeus* was also included in the analysis as another fossil sample.

Morphological character coding was based on the type species of the genera used, except for *Doryphoribius* and *Ramajendas* due to the lack of 18S rRNA sequences of their type species (Data S2). When possible, studies that provide the most recent redescriptions of the different species and high-quality microscope images were used as references for determining the character states. If not available, references of the original species descriptions were used, together with references containing high quality microscope images of the type species, and monographs of descriptions of eutardigrade genera (for the full list of references used, see Data S1). For the molecular dataset, the 18S rRNA sequences was aligned using MAFFT 7.4 [4] using the L-INS-i algorithm. The alignment was then visualized and both ends were manually trimmed using Aliview 1.28 [5] which resulted in a final length of 1774 nucleotide sequences (Data S3). Both datasets were then concatenated using Seaview 5.0 [6].

To assess the phylogenetic relationships of the samples, the resulting matrix (1810 characters: 36 morphological + 1774 molecular) was subjected to a Bayesian analysis using MrBayes 3.2 [7]. For the morphological set, the Mk model [8] + Gamma with the coding set to ‘variable’, which excluded two invariant characters was used. For the molecular set, the GTR model + Gamma +

proportion of invariable site (nst=6, rates=invgamma) was used, based on the best model scheme obtained using Partitionfinder 2.1 [9]. The analysis was run for 2 000 000 generations sampling every 500 generations and with a 25% burn-in frequency. Two runs were simultaneously done with each having one cold and three heated chains. Convergence was assessed by checking that the average deviation of split frequencies of the two runs were less than 0.01, effective sample size values were greater than 200 and the potential scale reduction factor was approximately = 1. A 50% majority rule consensus tree was then obtained to summarize the resulting analysis.

B. Divergence Time Estimation

To test the effect of using the Canadian fossil as a calibration point for its newly recovered taxonomic groupings, divergence times were estimated. The analyses used different combinations of two datasets – phylogenomics and 18S/28S rRNA barcodes with different sampling sizes. For each analysis, three different fossil node calibration strategies were used: 1) no tardigrade fossils were used with time estimation relying only on the root calibration (0fossil), 2) using *Mil. swolenskyi* as the sole calibration point for the entire crown-group of tardigrades (1fossil), and 3) using *Mil. swolenskyi* as a calibration point for the crown-group eutardigrades and *Beo. leggi* for the clade corresponding to the superfamily Hypsibioidea (2fossil). An additional strategy was done for the 18S/28S rRNA dataset similar to the third strategy but using *Beo. leggi* as a calibration point for the clade corresponding to the family Hypsibiidae (within Hypsibioidea) (also considered 2fossil). For this fourth calibration strategy, *Mixibius* was not included in the calibrated hypsibiid clade since it does not have *Hypsibius*-type external claws and its ambiguous position within hypsibioid families in recent molecular phylogenetic analyses [2,10]. Additionally, with the redescription of *Acutuncus* and subsequent erection of the new family Acutuncidae, there is no clear distinguishing characters between *Acutuncus* and *Mixibius* [10]. Complete details about the list of species included in the calibrated clades and calibration ages are in Table S3 and S4.

i. Phylogenomic Dataset

For the phylogenomic dataset, translated gene sequences from nine tardigrades representing all four major tardigrade groups (Arthotardigrada: *Actinarctus doryphorus*; Echiniscoidea: *Echiniscoides cf. sigismundi, Echiniscus testudo;* Apochela: *Milnesium* cf. *tardigradum;* Parachela: *Ramazzottius varieornatus, Hypsibius exemplaris, Richtersius* cf. *coronifer,*

Mesobiotus philippinicus, and *Paramacrobiotus* cf. *richtersi*) and one euarthropod (*Drosophila melanogaster*) as an outgroup were used. Aside from *Actinarctus doryphorus,* all the tardigrade assemblies were obtained from Mapalo et al., 2020 [11]. The protein sequences of *Drosophila melanogaster* (Release 6 plus ISO1 MT) were obtained from NCBI (https://www.ncbi.nlm.nih.gov/genome/?term=txid7227).

For *Actinartus doryphorus*, raw genomic reads from Howard et al., 2022 [13], were downloaded (PRJNA802334). After trimming using TrimGalore 0.5 (https://github.com/FelixKrueger/TrimGalore) to remove adapter and low-quality reads using default settings, the genome was assembled using SPAdes 3.8 [14] and assembly statistics were checked using Quast 5.2 [15]. Scaffolds less than 1000 bp were then removed using the filtercontigs.py script from GitHub (https://github.com/tinybio/filter_contigs) and the assembly statistics were rechecked using Quast (Table S7). Contaminants were checked using Blobtools 1.0 [16], removing raw reads corresponding to prokaryotes (i.e., bacteria and archaea), fungi, and viruses using the filter option. This produced a new set of raw reads that was used to assemble the genome again using SPAdes. The process of contig filtering, contamination removal, and genome assembly was repeated until the contaminants were approximately less than 1% (Figure S14), requiring three rounds.

Once contaminants were removed, soft-masking was done using RepeatMasker (http://www.repeatmasker.org) before evidence-based gene prediction was done using Augustus 3.3 [17]. Databases of all ecdysozoans present in the Augustus library were used (*Acyrthosiphon pisum, Aedes aegypti, Brugia malayi, Caenorhabditis elegans, Drosophila melanogaster, Nasonia vitripennis, Tribolium castaneum*) which resulted into seven individual files. The translated protein sequences were obtained using the "getAnnoFasta.pl" option of Augustus. All files were then concatenated, and all redundant sequences were removed using CD-Hit 4.6 [18] with the 100% option. The completeness of the assembly was assessed using BUSCO 5 [19] against the eukaryote and metazoan databases (Table S8). Lastly, the resulting file were subjected again to CD-Hit with 95% option to remove closely related sequences.

Gene homology searches between all the transcriptomes were done using OMA 2.1 [20]. After selecting genes that have at least 90% taxa occupancy, 335 orthologs were obtained. The 'selectslice.py' script used for the gene selection was from Schwentner et al., 2018 [21]. Even though the *Actinarctus* genome had a very low percent completeness (9.4%), we believe that the high

number of genes obtained justified the inclusion of this sample in our analyses. The 335 genes were then aligned using MAFFT using the "auto" option and concatenated using Phyutility [22]. This resulted in a matrix with a length of 139,117 amino acid sites (Data S4).

To determine the tardigrade topology, gene trees were first obtained from each of the 335 aligned gene homologs using IQTree 1.6 [23]. All the gene trees were then concatenated, and the resulting matrix was used as an input for ASTRAL 4.10 [24]. Divergence time estimates were calculated using the approximate likelihood method in MCMCTree [25] in the PAML 4.9 package [26] using the independent clock rate model with Birth-Death (BD) tree model. The sampling size parameter used was 0.003, which correspond to the total number of tardigrade species estimated in Bartels et al., 2016 [27]. For all the analyses, the substitution model used was the LG model with gamma rate distribution (alpha=0.5, no. of categories =4). Substitution rates were first estimated using CodeML in the PAML package and the results (mean=0.9857, var=0.6471) were used to set the values in the 'rgene_gamma' (a=1.5, b=1.52). Node calibration was done using uniform distribution for the age priors. Two runs were done for each fossil calibration strategy. Each run has a burn-in value of 200,000 iterations and sampling every 10,000 iterations until 10,000 samples are obtained. Convergence was assessed by plotting the time estimates from the two runs and was confirmed if their R^2 values was ~1 (Data S5). Since both runs showed comparable values, only the values from one run were used and shown in the succeeding results. To assess the effects of the priors, two runs without using the data were also done. This was done for each fossil strategy. All trees were visualized using Figtree 1.4 (http://tree.bio.ed.ac.uk/software/figtree/).

ii. 18S/28S rRNA Dataset

For the 18S/28S rRNA dataset, 139 tardigrades representing all tardigrade orders were used. When possible, each genus is represented by two species and the samples selected must have at least an 18S rRNA sequence. For the 28S rRNA, only overlapping sequences corresponding to one region of 28S was used. As a result of these selection process, 139 sequences of 18S rRNA and 80 sequences of 28S rRNA were used (Data S6). One euarthropod (*D. melanogaster*) was used as an outgroup for all analyses. Each rRNA sequences were individually aligned using MAFFT 7.490 with the L-INS-i algorithm. The alignments were then visualized and both ends were manually trimmed using Aliview 1.28 which resulted in a final length of 2166 and 1044 nucleotide sequences

for the 18S and 28S sequences, respectively. Both datasets were then concatenated using Seaview 5.0.4 resulting in a dataset with a length of 3210 nucleotide sequences (Data S7).

The tree topology was reconstructed using maximum likelihood (ML) and Bayesian inference (BI) using the best model scheme obtained from Partitionfinder 2.1. The ML tree was reconstructed using IQTree 1.6 [23] with the matrix divided into two partitions corresponding to each rRNA sequences, and the GTR+I+G model used for each partition. Bootstrap analysis was done using 1000 replicates, and the consensus tree was obtained using the default setting (i.e., extended majority-rule consensus). The BI tree was reconstructed using MrBayes 3.2. The matrix was partitioned according to the different rRNA sequences, and the GTR model + Gamma + proportion of invariable site (nst=6, rates=invgamma) for each partition was used. Additionally, the "statefreq", "revmat", "shape", "pinvar", and "tratio" were unlinked. The analysis was run for 10 000 000 generations sampling every 10,000 generations and with a 25% burn-in frequency. Two runs were simultaneously done with each having one cold and three heated chains. Convergence was assessed by checking that the average deviation of split frequencies of the two runs were less than 0.01, effective sample size values were greater than 200 and the potential scale reduction factor was approximately = 1. A 50% majority rule consensus tree was then obtained to summarize the resulting analysis.

Divergence time estimation was done using BEAST 2.6 [28] with the relaxed log normal clock model and BD tree model. The dataset was partitioned based on the type of rRNA sequences and BmodelTest [29] was used to select the substitution model for each partition. The clock and tree models were linked, with otherwise default settings. Node calibration was done using uniform distribution for the age priors. Each run lasted for 100,000,000 generations with sampling at every 10,000 generations and initial burn-in frequency of 2000. Convergence was assessed by checking the log files in Tracer 1.7 [30] and was confirmed if combined log files from the three runs showed ESS values greater than 200 for all statistics. For each fossil calibration strategy, three individual runs were done. Log and tree files from all three runs were combined, resampled at every 50,000 generations, and cleansed off the first 25% burn-in values using LogCombiner. The resulting log file was visualized in Tracer again to ensure that all statistics have ESS values greater than 200. Using the newly resampled tree file, TreeAnnotator from the BEAST package was used to obtain a maximum clade credibility using Common Ancestor (CA) heights as the node heights to produce

the final tree containing the divergence time estimates. For each calibration strategy, three runs of exclusive sampling from the priors were performed.

II. Systematic Paleontology

Phylum Tardigrada Doyère, 1840 [31]
Class Eutardigrada Richters, 1926 [32]
Order Parachela Schuster, Nelson, Grigarick and Christenberry, 1980 [33]
Superfamily Hypsibioidea Pilato, 1969 [34] in [35]
Family Hypsibiidae Pilato, 1969 [34]
Genus *Beorn* Cooper, 1964

Emended Diagnosis: Tardigrade with *Hypsibius*-type claws (i.e., external and internal claws (posterior and anterior claws) greatly differ in size and shape; claws with the secondary branch forming a continuous curve with its basal section). External and posterior claws with a primary branch connected to the basal section with an evident flexible part while internal and anterior claws are robust and rigid. Pseudolunules not observed. Accessory points present, at least on the posterior claw of the fourth pair of legs. Peribuccal and cephalic appendages not observed. Cuticle smooth.

Type species: *Beorn leggi* Cooper, 1964

Type locality: Secondary deposits along the beaches near the entrance of the Saskatchewan River into Cedar Lake, Canada [36]. Collected in 1940.

Type material: MCZ PALE-5213, holotype and only known specimen. The fossil is embedded in amber together with another smaller tardigrade (see below, Fig S1). The specimen is deposited in the Entomology Collection of the Museum of Comparative Zoology, Harvard University.

Diagnosis: As for genus.

Description: See main manuscript for full morphological description.

Remarks: *Beorn leggi* was originally described by having a well-defined cuticular frontal element that delineates the head and a body that is annulated by transverse lines. The images that we obtained reveal that these characteristics are not authentic, and the lines most likely correspond to cuticular folds produced during preservation (Fig 1). Our results show that the legs have the typical eutardigrade lobopodous leg instead of the telescopic legs found in some heterotardigrades. This misinterpretation might have been caused by some legs being contracted and producing cuticular folds along the legs. Our results also show that the specimen possesses *Hypsibius*-type claws (i.e., the external, or posterior, claws with the secondary branch forming a continuous curve with its basal section and the primary branch connected with an evident flexible part) in all legs (Fig 2,S2-S3). The apical cuticular projection (or spine) described to be on the anterior portion of the claws potentially corresponds to the cuticular extensions of the legs that covers the proximal portion of the claw (Fig 2B, arrow). Thus, the previously described characteristic of the claws (i.e., having the major ramus [primary branch] of its internal claws longer than the major ramus [primary branch] of its external claws] are also misinterpretations. Overall, the distinguishing characteristics of the genus that allowed the formalization of a new family are all negated by the newer high-quality images that we obtained. These results lead us to place *Beo. leggi* in the family Hypsibiidae and abolish the family Beornidae. Nevertheless, we did not abolish the genus (see discussion in main manuscript).

Superfamily Hypsibioidea Pilato, 1969 [34] in [35]

Genus *Aerobius* gen. nov. (Three-letter acronym: *Aer*)

Zoobank:http://www.zoobank.org/urn:lsid:zoobank.org:pub:E407CAA2-4928-4670-AB33-F4E5E9E4A589

Etymology: From the word "*aero*" or relating to air; due to the fossil appearing to be floating on air as it is embedded in the amber.

Diagnosis: Tardigrade with a modified *Isohypsibius*-type claws (i.e., external and internal claws greatly differ in shape but slightly similar in size; external claws with the secondary branch and the basal section form a right angle, but with the primary branch connected with an evident flexible part, characterized by a curved based of the primary branch) in legs I-III (most evident in claw II),

and possibly *Hypsibius*-type claws (i.e., posterior and anterior claws greatly differ in shape and size; claws with the secondary branch forming a continuous curve with its basal section) or a modified *Isohypsibius*-type claws (i.e., external and internal claws greatly differ in shape but slightly similar in size; external claws with the secondary branch and the basal section form a right angle, but with the primary branch connected with an evident flexible part, characterized by a curved based of the primary branch) in legs IV. Posterior claw with a primary branch connected to the basal section with an evident flexible part and length notably longer than the secondary branch. Internal and anterior claws appear to be robust and rigid. Accessory points and pseudolunules present, at least in the internal claws. Elliptical organ could be present. Peribuccal and cephalic appendages not observed. Cuticle smooth.

Type species: *Aerobius dactylus* sp. nov.

Etymology: From the word "*dactylo*" or finger; for the unique combination of morphological characteristics of the claw or "fingers" of the fossil.

Type locality: Secondary deposits along the beaches near the entrance of the Saskatchewan River into Cedar Lake, Canada [36]. Collected in 1940.

Type material: MCZ PALE-45862, holotype and only known specimen. the fossil is embedded in amber together with *Beorn leggi*. The specimen is deposited in the Entomology Collection of the Museum of Comparative Zoology, Harvard University.

Diagnosis: As for genus.

Description: See main manuscript for full morphological description.

Remarks: The smaller tardigrade synclusion was suggested to be a juvenile heterotardigrade due to the perceived presence of a lateral cirrus, clava, and two claws on one leg. Our recent images did not support these observations. In fact, given the claw morphology and lack of cephalic appendages, this tardigrade is undeniably a eutardigrade (Fig 3,S4). The fossil has modified

Isohypsibius-type claws in legs I-III (most evident in claw II), but our images do not allow us to distinguish whether the claws in legs IV are *Hypsibius*-type claws or modified *Isohypsibius*-type claw (Fig 4,S4). Regardless of the morphology of the claws in the hind legs, the combination of claw morphology between legs I-III and legs IV are still not present in any extant tardigrade genus. Some hypsibioids (i.e., *Acutuncus* and *Mixibius*) also possess *Isohypsibius*-type claws, but this claw type is different from what is observed in this fossil [10,37,38]. A circular structure was observed in the cephalic region which could be elliptical organs (Fig S5). This structure is only currently observed in two hypsibioid families (i.e., calohypsibiids, ramazzottiids) that possess claw morphologies that are different from the fossil [38–40]. However, the current images is not enough to confirm its validity as an elliptical organ. Regardless, the unique combination of claw characters, we attribute this specimen to a new genus and species, *Aerobius dactylus* gen. et sp. nov., to account for its distinct apomorphies. It should be noted that new tardigrade genera have been erected before by the basis of their claw morphologies being different from other extant tardigrades [41].

Even though the total-evidence based phylogenetic tree can only place *Aer. dactylus* gen. et sp. nov. within the superfamily Hypsibioidea (Fig 5), we hypothesize that it may be closely related to the family Acutuncidae. Species of this extant family (i.e., *Acutuncus*) also have *Isohypsibius*-type external claws [10], but they lack the evident flexible connection between the primary branch and basal section observed *in Aer. dactylus* gen. et sp. nov.

Supplementary Figures

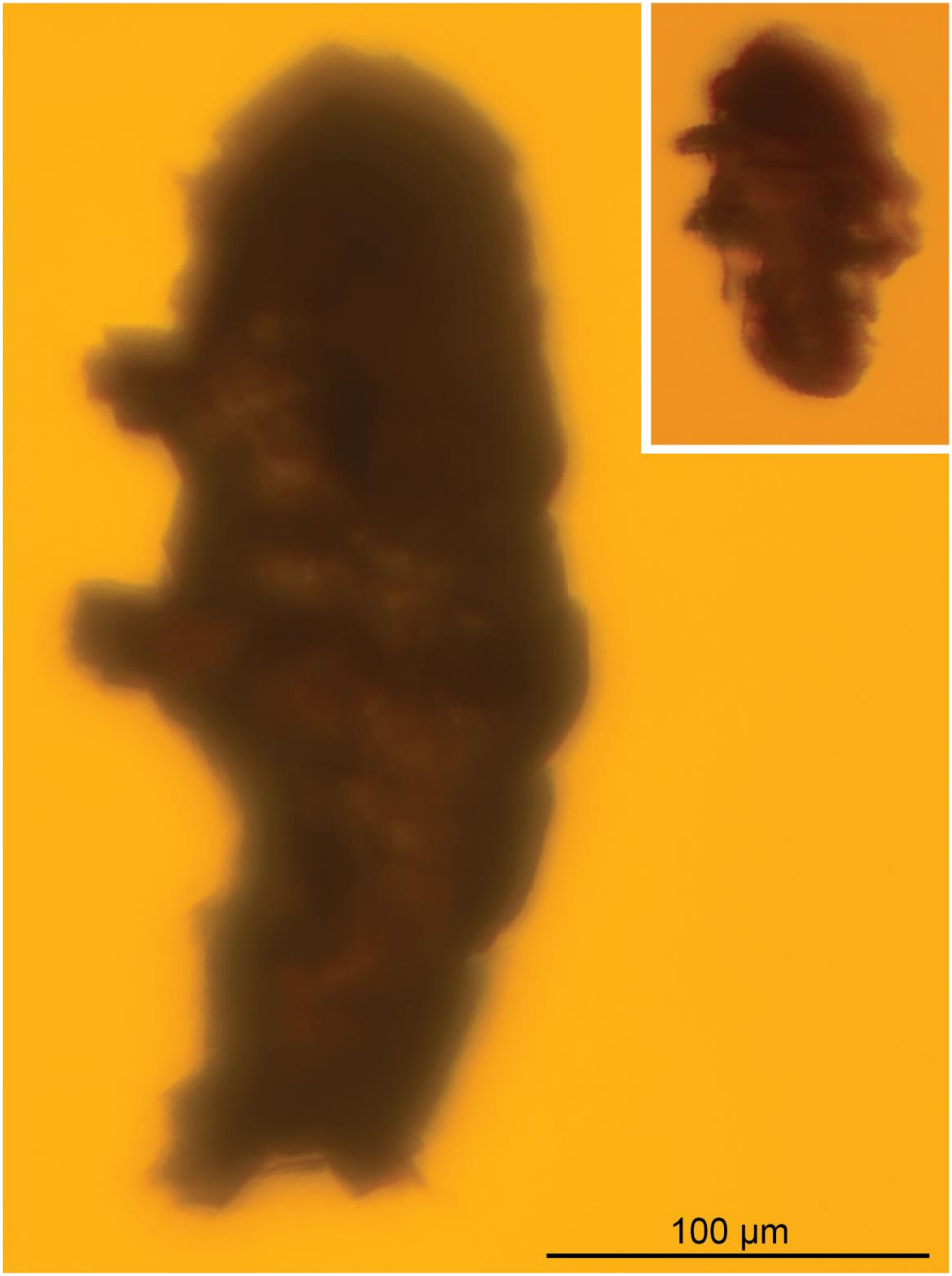

Fig S1. *Beorn leggi* and *Aerobius dactylus* gen. et sp. nov size comparison. *Beorn leggi* is ~300 um while *Aerobius dactylus* (inset) is ~100 um.

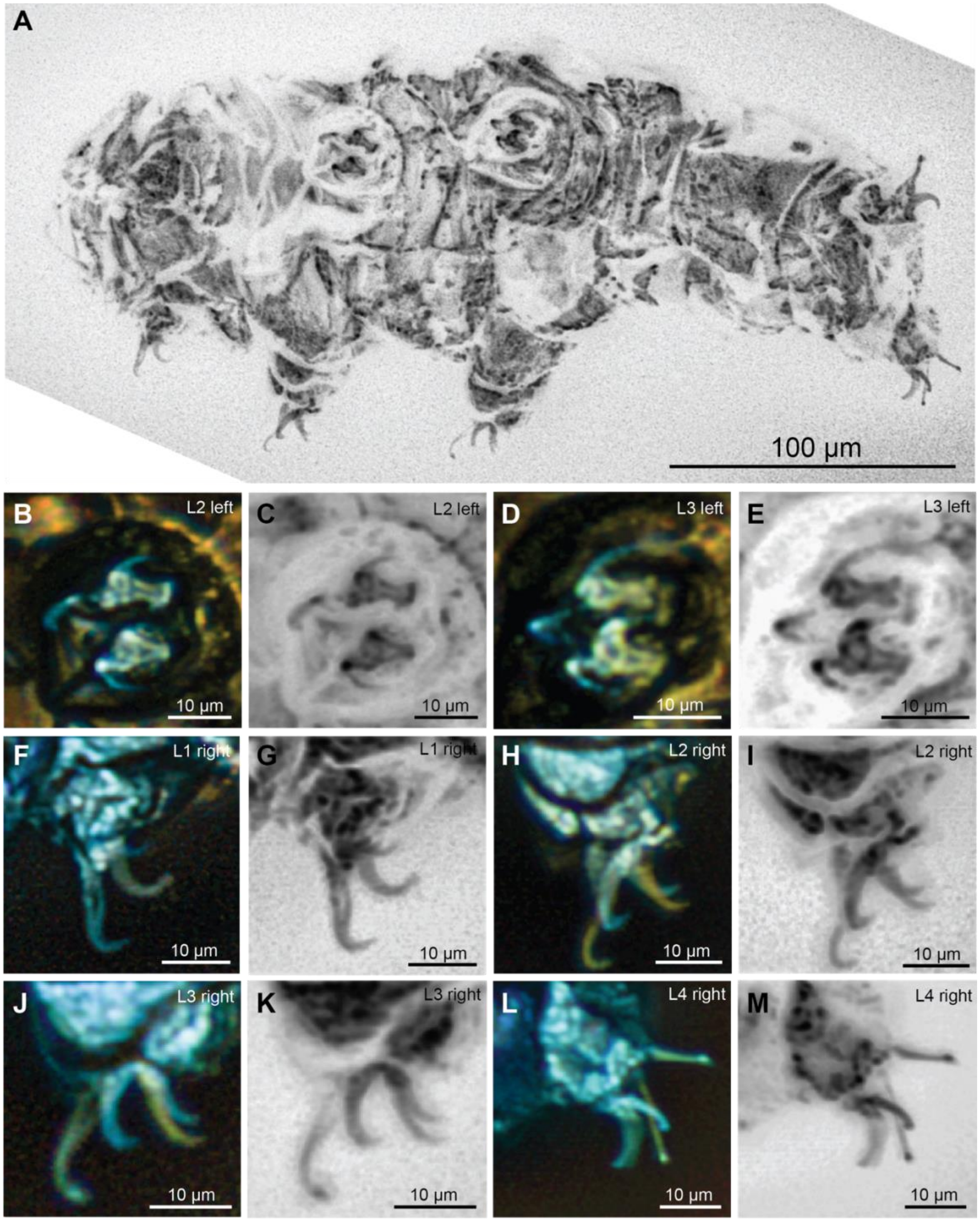

Figure S2. Ventral view of *Beorn leggi* photographed with autofluorescence under confocal microscope at 639 nm. (A,C,E,G,I,K,M) Specimen and claws viewed in inverted greyscale to highlight autofluorescence intensity (darker–more intense, lighter–least intense). (B,D,F,H,J,L) Claws viewed in different colors indicating z-depth, with violet to red gradient representing the shallowest to deepest planes, respectively. Ln – leg number.

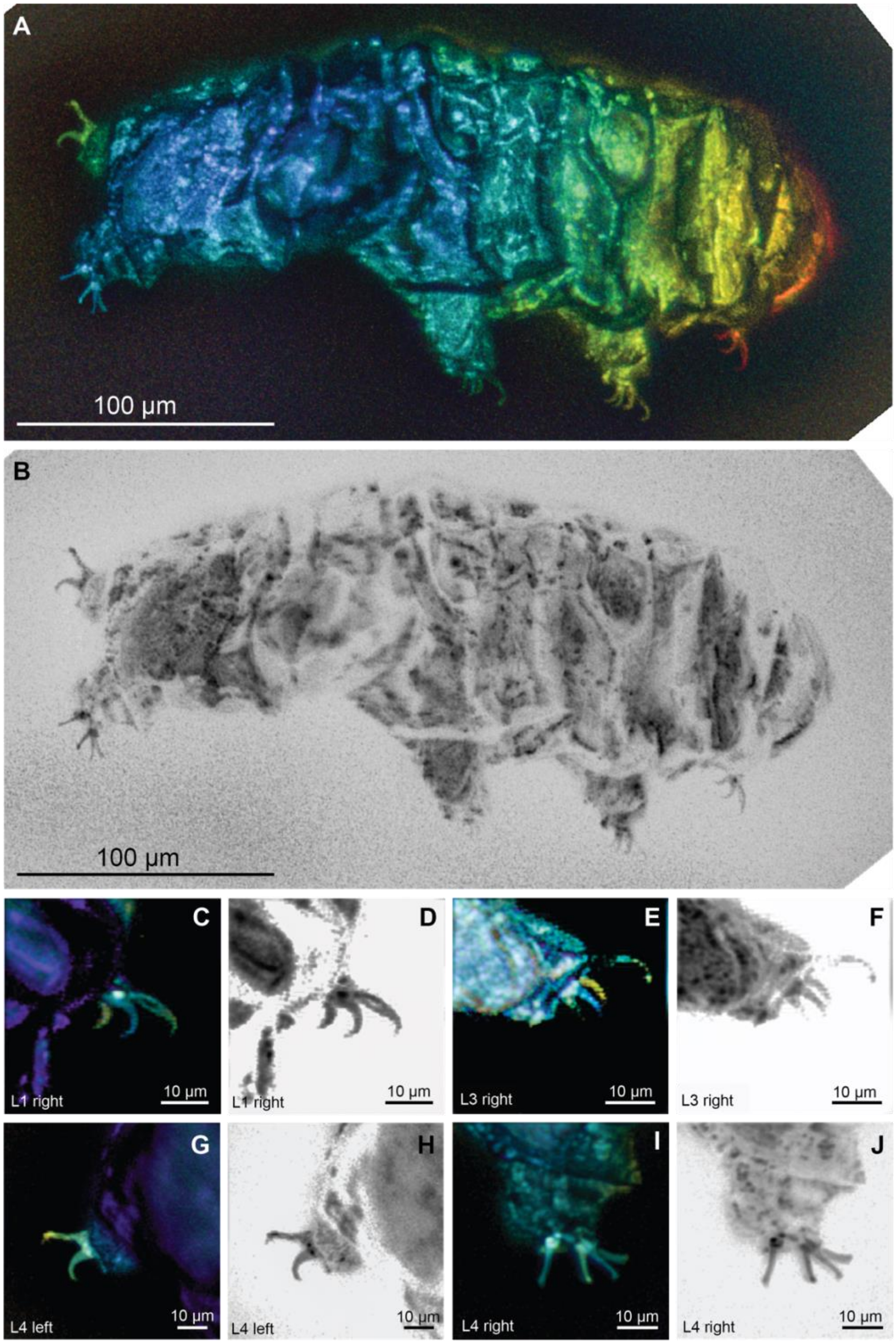

Figure S3. Dorsal view of *Beorn leggi* (A,C,E,G,I) Specimen and claws photographed with autofluorescence under confocal microscope at 639 nm; different colors indicate z-depth, with violet to red gradient representing the shallowest to deepest planes, respectively. (B,D,F,H,J) Specimen and claws viewed in inverted greyscale to highlight autofluorescence intensity (darker–more intense, lighter–least intense). Ln – leg number.

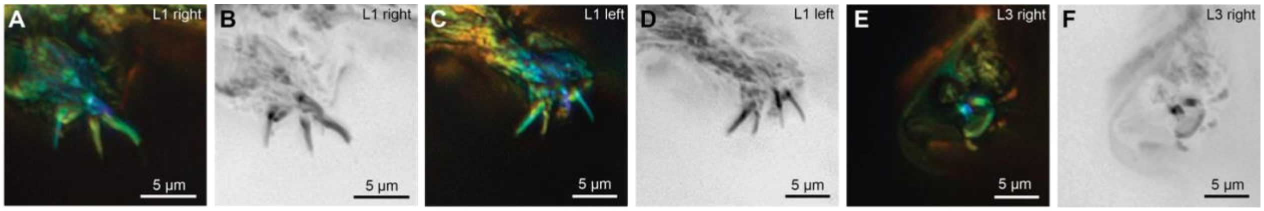

Figure S4. Claws of *Aerobius dactylus* gen. et sp. nov. (A,C,E) Specimen and claws photographed with autofluorescence under confocal microscope at 639 nm; different colors indicate z-depth, with violet to red gradient representing the shallowest to deepest planes, respectively. (B,D,F) Specimen and claws viewed in inverted greyscale to highlight autofluorescence intensity (darker–more intense, lighter–least intense). Ln – leg number.

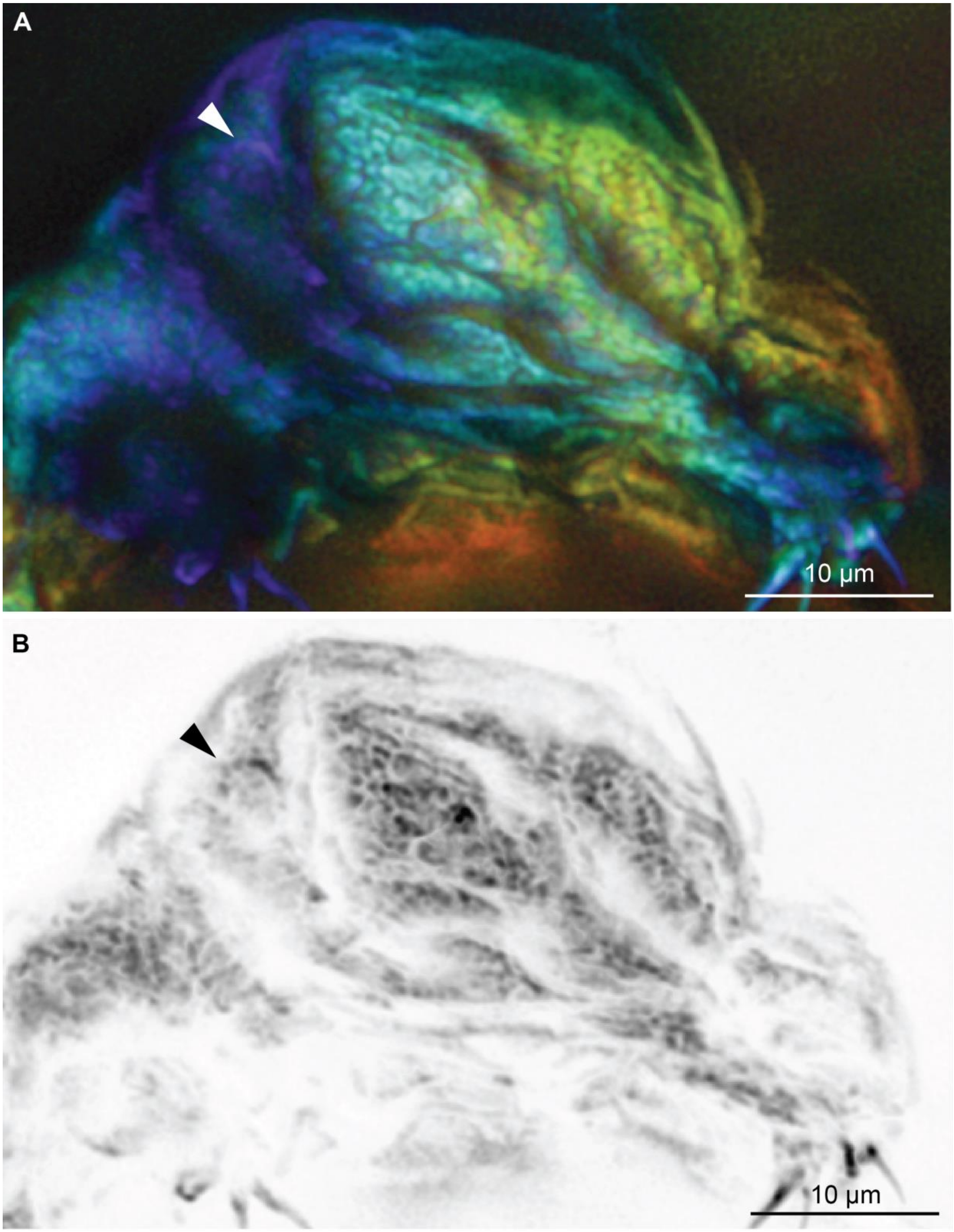

Figure S5. Ventral view of *Aerobius dactylus* head region. Arrow heads point to a structure that could be an elliptical organ.

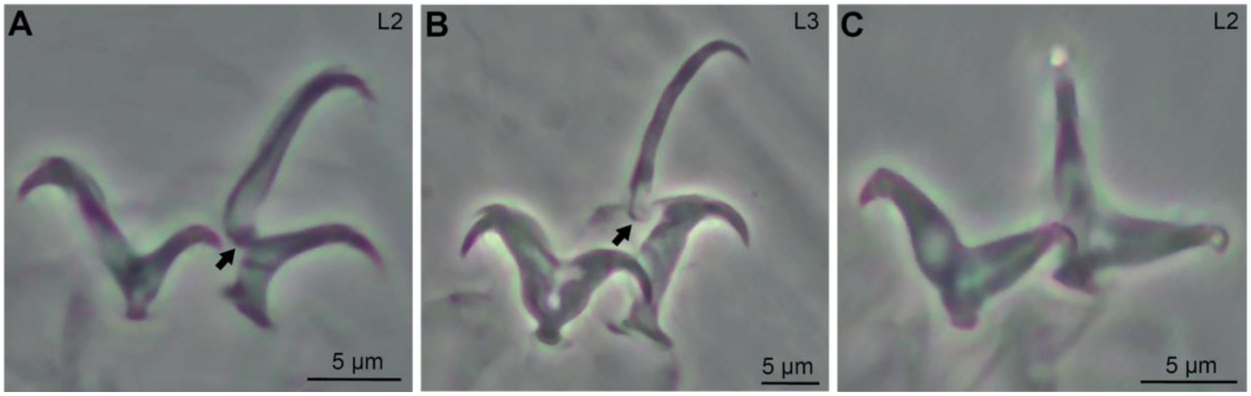

Figure S6. Claws of extant tardigrades. (A) *Hypsibius*-type, (B) *Ramazzottius*-type, (C) *Isohypsibius*-type external claws. Arrow indicates the flexible connection between the basal section and the primary branch. Ln – leg number.

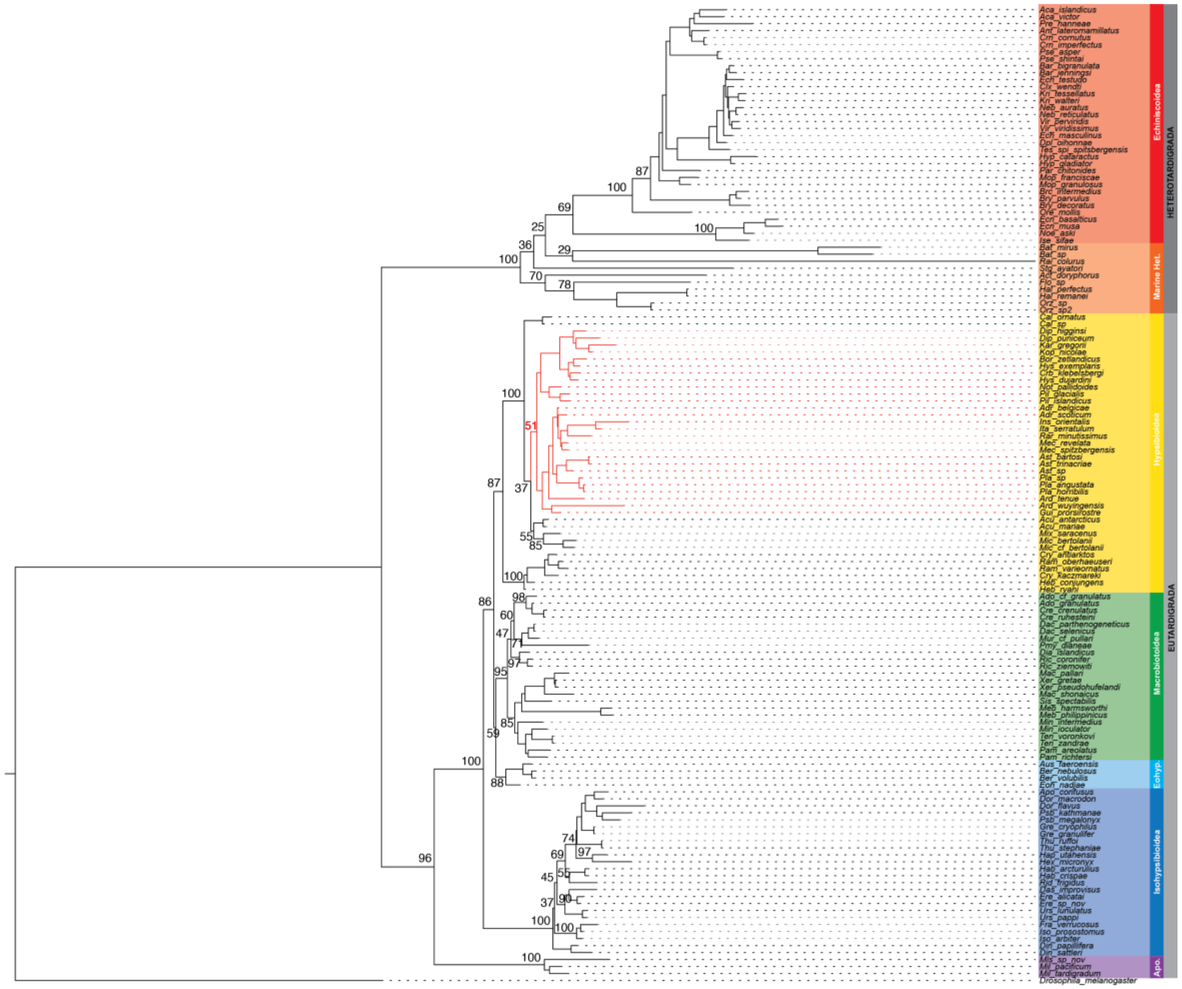

Figure S7. Phylogenetic result of the maximum likelihood using IQTree and the 18S/28S rRNA dataset. Values above or below the node represent the bootstrap support and shown only until the family level, with at least two species representing the family. The clade highlighted in red corresponds to the family Hypsibiidae *sensu* Bertolani et al., (2014) [2]. Apo. – Apochela; Eohyp. – Eohypsibioidea; Marine Het. – marine Heterotardigrada (formerly Arthrotardigrada).

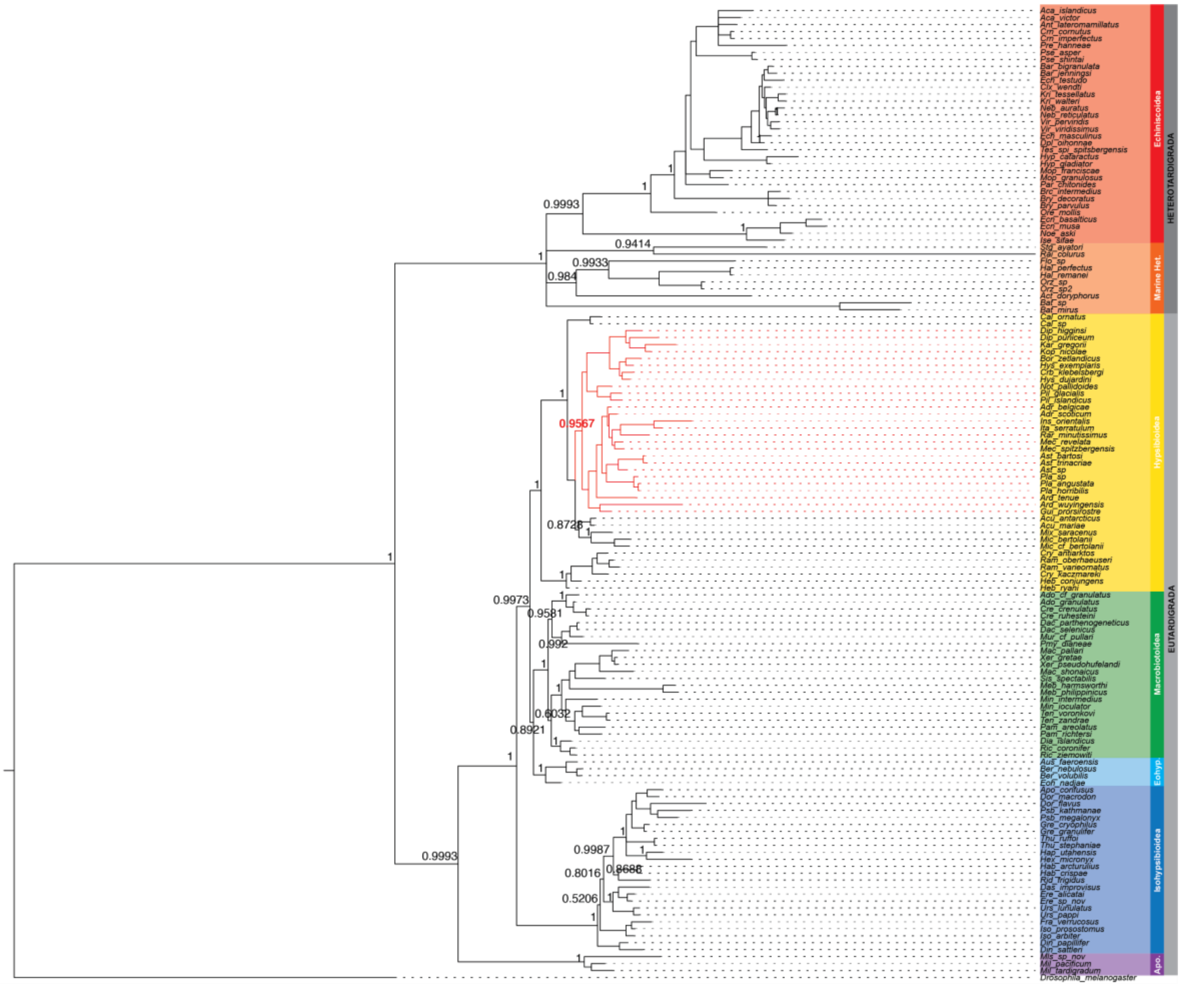

Figure S8. Phylogenetic result of the Bayesian inference using MRBayes and the 18S/28S rRNA dataset. Values above or below the node represent the posterior probability values and shown only until the family level, with at least two species representing the family. The clade highlighted in red corresponds to the family Hypsibiidae *sensu* Bertolani et al., (2014) [2]. Apo. – Apochela; Eohyp. – Eohypsibioidea; Marine Het. – marine Heterotardigrada (formerly Arthrotardigrada)

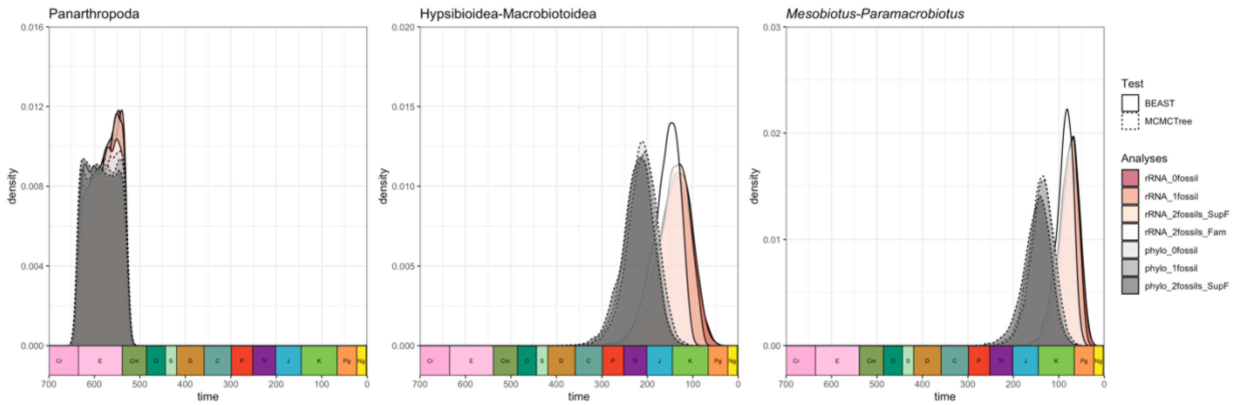

Figure S9. Density plots of divergence time estimates of different tardigrade clades. Different colors represent different calibration strategies (0fossil – 1st strategy, 1fossil – 2nd strategy, 2fossils_SupF – 3rd strategy, 2fossils_Fam – 4th strategy) and dataset used (rRNA – 18S/28S rRNA, phylo – phylogenomic). Line type represent the type of analyses used (i.e., BEAST or MCMCtree).

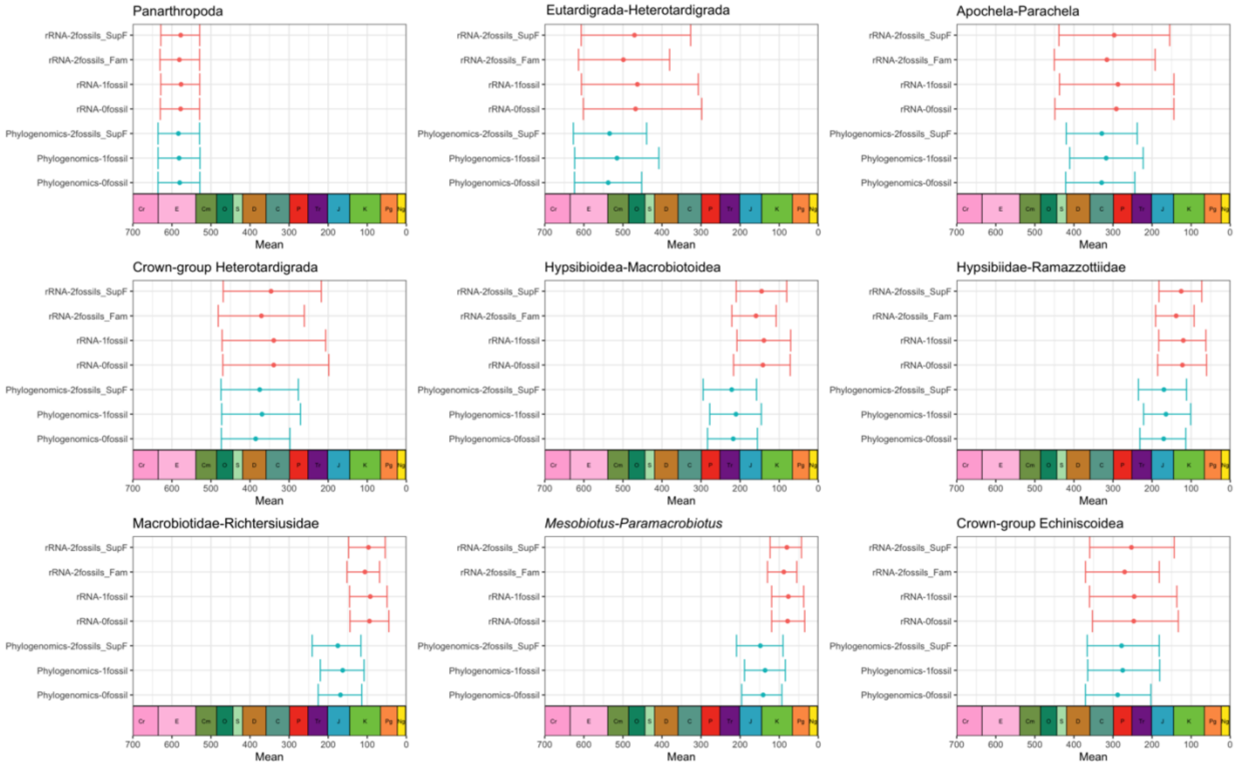

Fig S10. Divergence time estimates of different tardigrade clades obtained using the phylogenomics and the 18S/28S rRNA datasets. Dot represents the mean CA value while the error bars mark the minimum and maximum boundary of the 95% height posterior density. Different colors represent different programs used – blue: MCMCtree; red: BEAST.

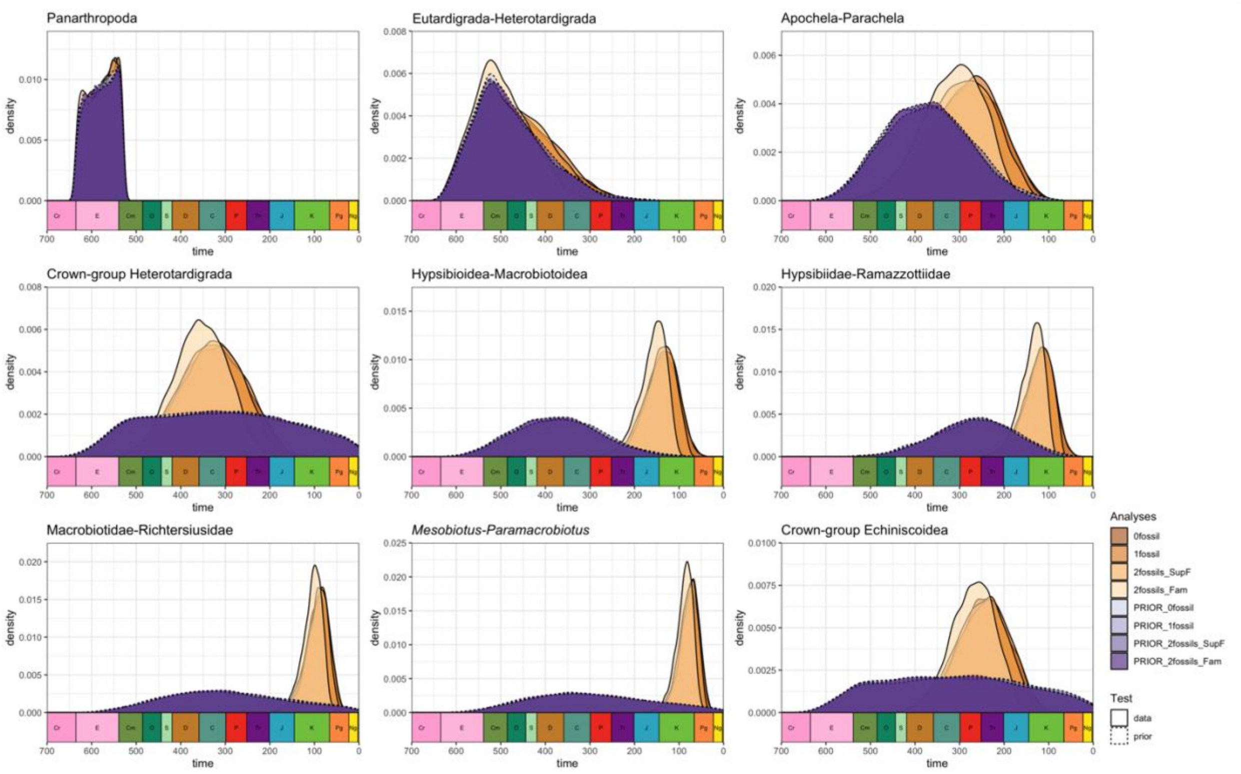

Figure S11. Density plots of divergence time of different tardigrade clades estimated using BEAST and the 18S/28S rRNA dataset. Different colors represent different calibration strategies (0fossil – 1st strategy, 1fossil – 2nd strategy, 2fossils_SupF – 3rd strategy, 2fossils_Fam – 4th strategy) and dataset used (with data or prior-only). Line type represent the type of dataset used (i.e., with data or prior-only)

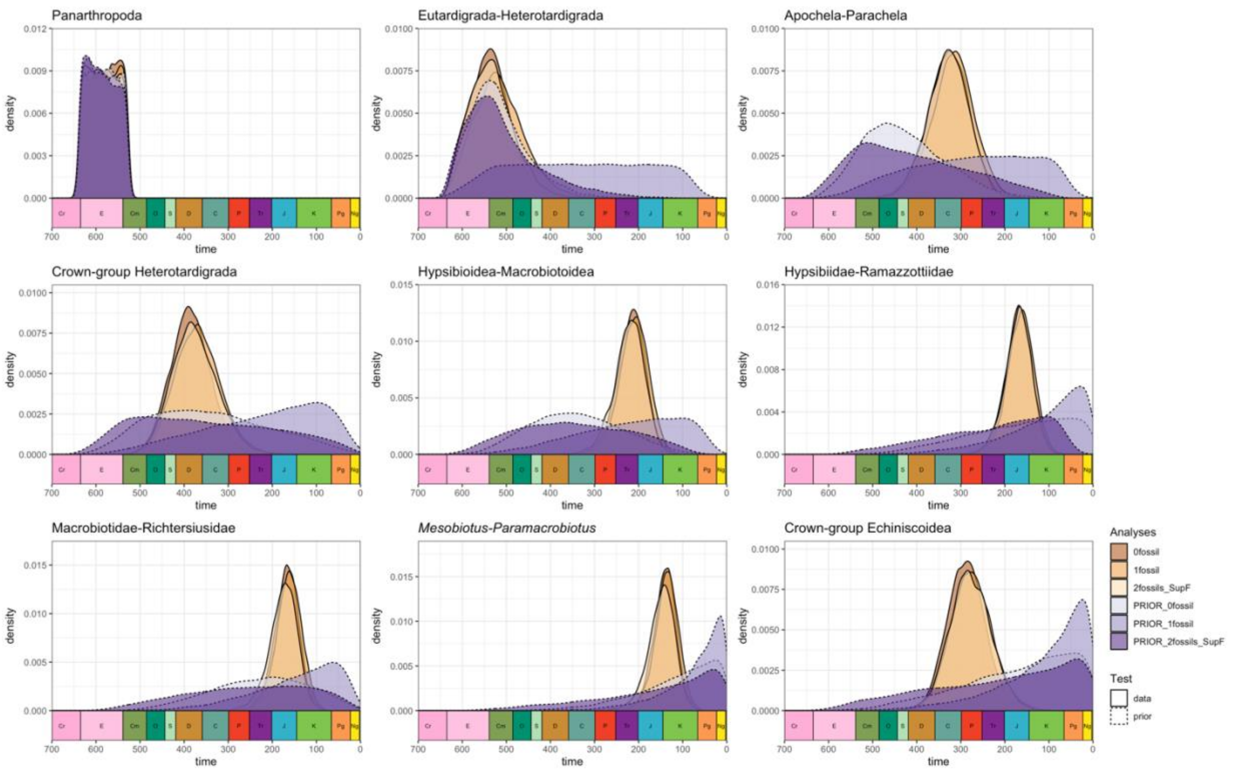

Figure S12. Density plots of divergence time of different tardigrade clades estimated using MCMCTree and the phylogenomic dataset. Different colors represent different calibration strategies (0fossil – 1st strategy, 1fossil – 2nd strategy, 2fossils_SupF – 3rd strategy, 2fossils_Fam – 4th strategy) and dataset used (with data or prior-only). Line type represent the type of dataset used (i.e., with data or prior-only)

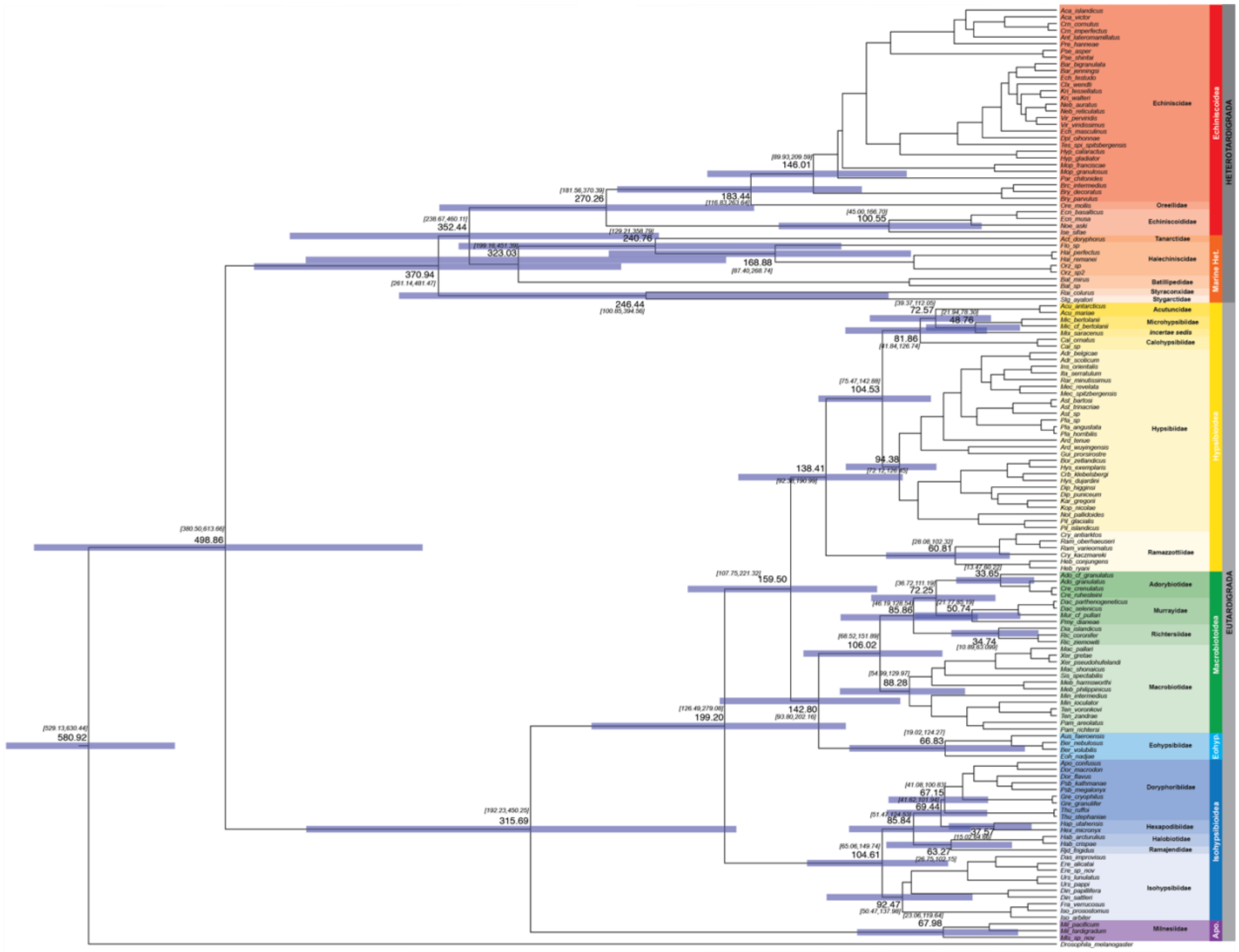

Figure S13. Divergence time estimates of major tardigrade groups obtained using BEAST and the 18S/28S rRNA dataset with *Beorn leggi* used to calibrate at the family level. Values above the node represents the mean CA value. Node bars represent the range of the 95% height posterior density (HPD) with the values of the minimum and maximum boundary of the 95% HPD listed above the mean in italicized text. Node bars are shown only until the family level, with at least two species representing the family. Apo. – Apochela; Eohyp. – Eohypsibioidea; Marine Het. – marine Heterotardigrada (formerly Arthrotardigrada).

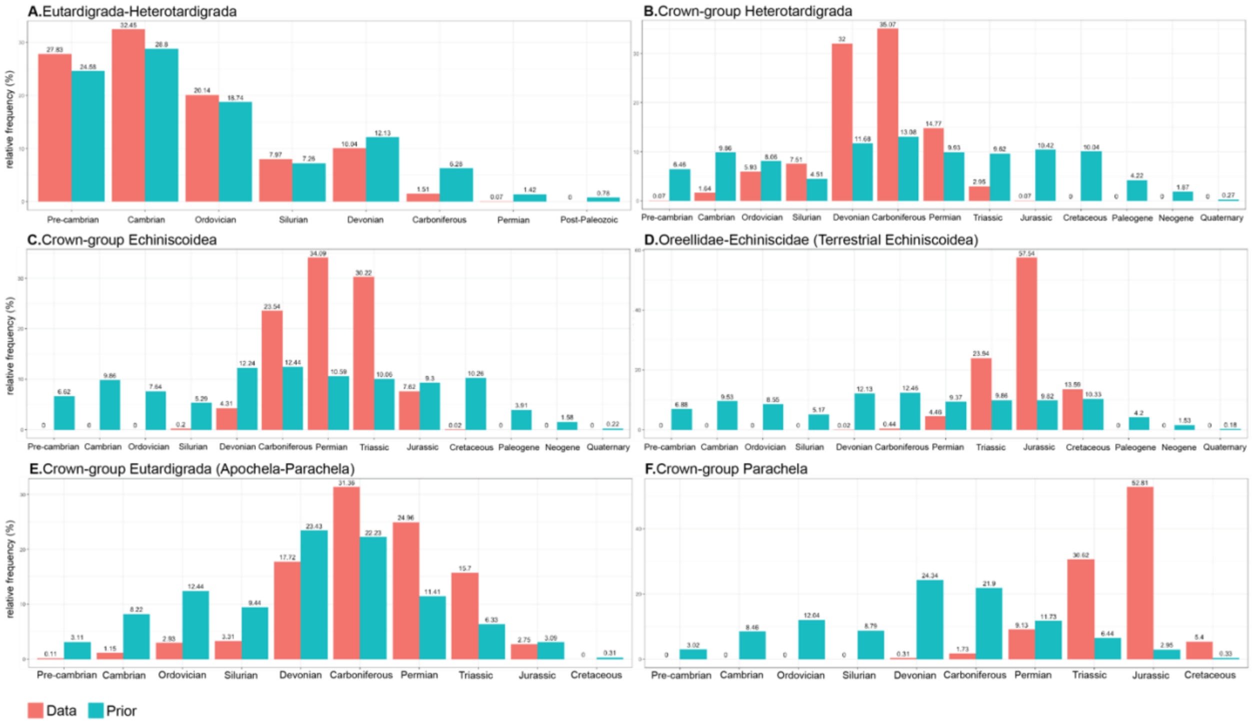

Figure S14. Relative frequencies of clade divergence time estimates (with respect to their corresponding geological time periods) obtained from the posterior samples of 4503 trees using BEAST and the 18S/28S rRNA dataset with *Beorn leggi* used to calibrate at the family level. The estimated age of a clade from each tree from the posterior tree samples were extracted and their corresponding geological time periods were assigned. This provided information on how often a geological time period is estimated to be the time of divergence of a clade of interest.

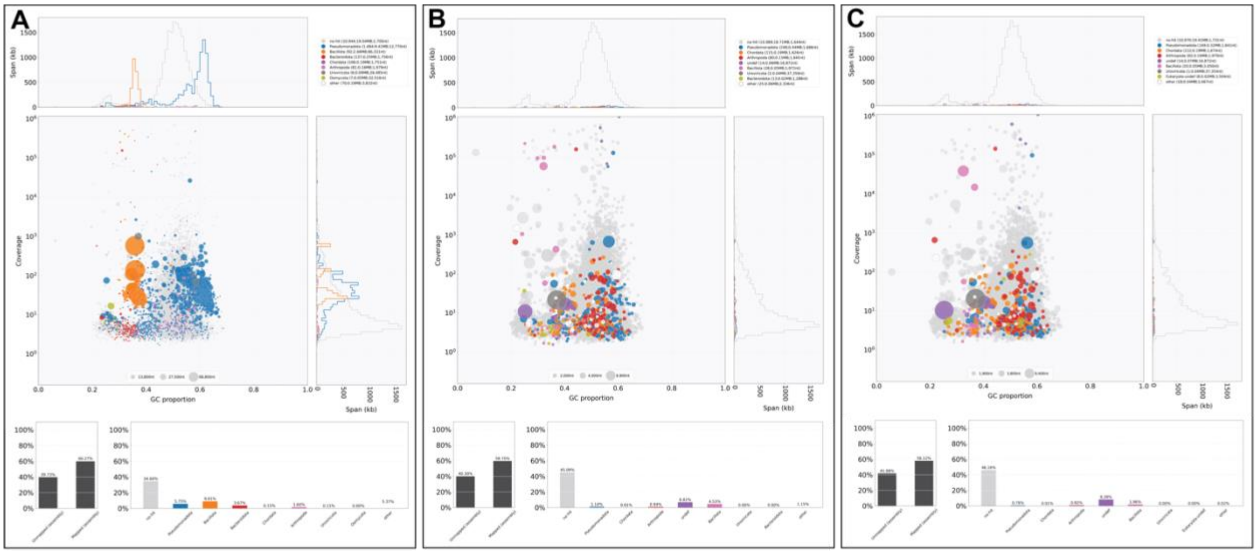

Figure S15. Blobtools results showing the degree of contaminations present in the assembly. Assembled genome pre-contamination removal (A), first round of contamination removal (B), and second round of contamination removal (C).

Supplementary Table

Table S1. Genbank accession numbers of the 18S rRNA sequences used for the total-evidence phylogenetic analysis.

Order	Superfamily	Family	Species	Accession Number
Apochela	-	Milnesiidae	*Milnesium tardigradum*	MG912554
Parachela	Eohypsibioidea	Eohypsibiidae	*Eohypsibius nadjae*	HQ604921
Parachela	Hypsibioidea	Calohypsibiidae	*Calohypsibius ornatus*	MH279652
Parachela	Hypsibioidea	Hypsibiidae	*Hypsibius dujardini*	MG777532
Parachela	Hypsibioidea	Microhypsibiidae	*Microhypsibius bertolanii*	HQ604991
Parachela	Hypsibioidea	Acutuncidae	*Acutuncus antarcticus*	OM278641
Parachela	Hypsibioidea	Ramazzottiidae	*Ramazzottius oberhaeuseri*	MG573241
Parachela	Isohypsibioidea	Doryphoribiiae	*Doryphoribius macrodon*	HQ604942
Parachela	Isohypsibioidea	Halobiotidae	*Halobiotus crispae*	EF620401
Parachela	Isohypsibioidea	Hexapodibiidae	*Hexapodibius micronyx*	MK675930
Parachela	Isohypsibioidea	Isohypsibiidae	*Isohypsibius prosostomus*	EF620404
Parachela	Isohypsibioidea	Ramajendidae	*Ramajendas frigidus*	MZ050453
Parachela	Macrobiotoidea	Adorybiotiidae	*Adorybiotus granulatus*	HQ604961
Parachela	Macrobiotoidea	Macrobiotidae	*Macrobiotus hufelandi*	GQ849024
Parachela	Macrobiotoidea	Murrayidae	*Murrayon pullari*	GQ849026
Parachela	Macrobiotoidea	Richtersiusidae	*Richtersius coronifer*	MH681760

Table S2. Fossil calibration strategies used in the MCMC analyses using the phylogenomic dataset. All tardigrade genera are listed using the three-letter abbreviations proposed in [42,43]

Strategy	Calibrated Node	Species	Age (Mya)	Age Reference
**1st strategy:** only root calibrated	Eurthropod-Tardigrade	Arthropod: *Drosophila melanogaster* Tardigrade: *Act. doryphorus, Ech. testudo, Ecn. cf. sigismundi, Mil. cf. tardigradum, Hys. exemplaris, Ram. varieornatus, Ric. cf. coronifer, Meb. philippinicus., Pam. cf. richtersi.*	Min: 528.82 Max: 636.1	[44]
**2nd strategy:** a. *Mil. swolenskyi* calibrating crown-group tardigrade	Eurthropod-Tardigrada	Similar to 1st strategy	Min: 528.82 Max: 636.1	[44]
	Crown-group Tardigrade	*Act. doryphorus, Ech. testudo, Ecn. cf. sigismundi, Mil. cf. tardigradum, Hys. exemplaris, Ram. varieornatus, Ric. cf. coronifer, Meb. philippinicus., Pam. cf. richtersi.*	Min: 89.8 Max: 636.1	Minimum age based on the upper boundary of the Turonian Age [45], which is the age of the New Jersey amber where *Mil. swolenskyi* is embedded [46,47]
**3rd strategy:** *a. Mil. swolesnkyi* calibrating crown-group eutardigrade *b, Beo. leggi* calibrating crown-group superfamily Hypsibioidea	Euarthropod-Tardigrada	Similar to 1st strategy	Min: 528.82 Max: 636.1	[44]
	Crown-group Eutardigrade	*Mil. cf. tardigradum, Hys. exemplaris, Ram. varieornatus, Ric. cf. coronifer, Meb. philippinicus., Pam. cf. richtersi.*	Min: 89.8 Max: 636.1	Minimum age based on the upper boundary of the Turonian Age [45], which is the age of the New Jersey amber where *Mil. swolenskyi* is embedded [46,47]
	Crown-group Hypsibioidea	*Hys. exemplaris, Ram. varieornatus,*	Min: 72.1 Max: 636.1	*Beo. leggi* is embedded in an amber from Cedar Lake, Canada. Ambers from this locality are correlated to the Grassy Lake ambers [48] which is dated around the Campanian Age [49]. Thus, the minimum age is based on the upper boundary of the Campanian Age [50]

Table S3. Fossil calibration strategies used in the BEAST analyses using the 18S/28S rRNA dataset. All tardigrade genera are listed using the three-letter abbreviations proposed in [42,43]

Strategy	Calibrated Node	Species	Age (Mya)	Age Reference
**1st strategy:** only root calibrated	***Eurthropod-Tardigrade***	Arthropod: *Drosophila melanogaster* Tardigrade: ***See LIST A***	Min: 528.82 Max: 636.1	[44]
	Crown-group Tardigrade	***See LIST A***	Not calibrated	-
	Crown-group Eutardigrade	***See LIST B***	Not calibrated	-
	Crown-group Hypsibioidea	***See LIST C***	Not calibrated	-
	Crown-group Hypsibiidae	***See LIST D***	Not calibrated	-
**2nd strategy:** a. *Mil. swolenskyi* calibrating crown-group tardigrade	***Eurthropod-Tardigrada***	Similar to 1st strategy	Min: 528.82 Max: 636.1	[44]
	Crown-group Tardigrade	Similar to 1st strategy	Min: 89.8 Max: 636.1	Minimum age based on the upper boundary of the Turonian Age [45], which is the age of the New Jersey amber where *Mil. swolenskyi* is embedded [46,47]
	Crown-group Eutardigrade	Similar to 1st strategy	Not calibrated	-
	Crown-group Hypsibioidea	Similar to 1st strategy	Not calibrated	-
	Crown-group Hypsibiidae	Similar to 1st strategy	Not calibrated	-
**3rd strategy:** *a. Mil. swolesnkyi* calibrating crown-group eutardigrade *b, Beo. leggi* calibrating crown-group superfamily Hypsibioidea	***Euarthropod-Tardigrada***	Similar to 1st strategy	Min: 528.82 Max: 636.1	[44]
	Crown-group Tardigrade	Similar to 1st strategy	Not calibrated	-
	Crown-group Eutardigrade	Similar to 1st strategy	Min: 89.8 Max: 636.1	Minimum age based on the upper boundary of the Turonian Age [45], which is the age of the New Jersey amber where *Mil. swolenskyi* is embedded [46,47]
	Crown-group Hypsibioidea	Similar to 1st strategy	Min: 72.1 Max: 636.1	*Beo. leggi* is embedded in an amber from Cedar Lake, Canada. Ambers from this locality are correlated to the Grassy Lake ambers [48] which is dated around the Campanian Age [49]. Thus, the minimum age is based on the upper boundary of the Campanian Age [50]
	Crown-group Hypsibiidae	Similar to 1st strategy	Not calibrated	-
**4th strategy:** *a. Mil. swolesnkyi* calibrating crown-group eutardigrade *b, Beo. leggi* calibrating crown-group family Hypsibiidae	***Eurthropod-Tardigrada***	Similar to 1st strategy	Min: 528.82 Max: 636.1	[44]
	Crown-group Tardigrade	Similar to 1st strategy	Not calibrated	-
	Crown-group Eutardigrade	Similar to 1st strategy	Min: 89.8 Max: 636.1	Minimum age based on the upper boundary of the Turonian Age [45], which is the age of the New Jersey amber where *Mil. swolenskyi* is embedded [46,47]
	Crown-group Hypsibioidea	Similar to 1st strategy	Not calibrated	-
	Crown-group Hypsibiidae	Similar to 1st strategy	Min: 72.1 Max: 636.1	*Beo. leggi* is embedded in an amber from Cedar Lake, Canada. Ambers from this locality are correlated to the Grassy Lake ambers [48] which is dated around the Campanian Age [49]. Thus, the minimum age is based on the upper boundary of the Campanian Age [50]

List A

Bat_mirus
Bat_sp
Flo_sp
Hal_perfectus
Hal_remanei
Orz_sp
Orz_sp2
Stg_ayatori
Rai_colurus
Act_doryphorus
Ecn_basalticus
Ecn_musa
Noe_aski
Ise_sifae
Ore_mollis
Aca_islandicus
Aca_victor
Ant_lateromamillatus
Bar_bigranulata
Bar_jenningsi
Brc_intermedius
Bry_decoratus
Bry_parvulus
Clx_wendti
Crn_cornutus
Crn_imperfectus
Dpl_oihonnae
Ech_masculinus
Ech_testudo
Hyp_cataractus
Hyp_gladiator
Kri_tessellatus
Kri_walteri
Mop_franciscae
Mop_granulosus
Neb_auratus
Neb_reticulatus
Par_chitonides
Pre_hanneae
Pse_asper
Pse_shintai
Tes_spi_spitsbergensis
Vir_perviridis
Vir_viridissimus
Mil_pacificum
Mil_tardigradum
Mls_sp_nov
Aus_faeroensis
Ber_nebulosus
Ber_volubilis
Eoh_nadjae
Cal_ornatus
Cal_sp
Dip_higginsi
Dip_puniceum
Kar_gregorii
Kop_nicolae
Bor_zetlandicus
Crb_klebelsbergi
Hys_dujardini
Hys_exemplaris
Adr_belgicae
Adr_scoticum
Ard_tenue
Ard_wuyingensis
Ast_bartosi
Ast_sp
Ast_trinacriae
Gui_prorsirostre
Ins_orientalis
Ita_serratulum
Mec_revelata
Mec_spitzbergensis
Pla_sp
Pla_angustata
Pla_horribilis
Rar_minutissimus
Not_pallidoides
Pil_glacialis
Pil_islandicus
Acu_antarcticus
Acu_mariae
Mix_saracenus
Mic_bertolanii
Mic_cf_bertolanii
Cry_antiarktos
Cry_kaczmareki
Heb_conjungens
Heb_ryani
Ram_oberhaeuseri
Ram_varieornatus
Apo_confusus
Dor_flavus
Dor_macrodon
Gre_cryophilus
Gre_granulifer
Psb_kathmanae
Psb_megalonyx
Thu_ruffoi
Thu_stephaniae
Hab_arcturulius
Hab_crispae
Hap_utahensis
Hex_micronyx
Das_improvisus
Din_papillifera
Din_sattleri
Ere_alicatai
Ere_sp_nov
Fra_verrucosus
Iso_arbiter
Iso_prosostomus
Urs_lunulatus
Urs_pappi
Rjd_frigidus
Ado_cf_granulatus
Ado_granulatus
Cre_crenulatus
Cre_ruhesteini
Mac_pallari
Mac_shonaicus
Meb_harmsworthi
Meb_philippinicus
Min_intermedius
Min_ioculator
Pam_areolatus
Pam_richtersi
Sis_spectabilis
Ten_voronkovi
Ten_zandrae
Xer_gretae
Xer_pseudohufelandi
Dac_parthenogeneticus
Dac_selenicus
Mur_cf_pullari
Pmy_dianeae
Dia_islandicus
Ric_coronifer
Ric_ziemowiti

List B

Mil_pacificum
Mil_tardigradum
Mls_sp_nov
Aus_faeroensis
Ber_nebulosus
Ber_volubilis
Eoh_nadjae
Cal_ornatus
Cal_sp
Dip_higginsi
Dip_puniceum
Kar_gregorii
Kop_nicolae
Bor_zetlandicus
Crb_klebelsbergi
Hys_dujardini
Hys_exemplaris
Adr_belgicae
Adr_scoticum
Ard_tenue
Ard_wuyingensis
Ast_bartosi
Ast_sp
Ast_trinacriae
Gui_prorsirostre
Ins_orientalis
Ita_serratulum
Mec_revelata
Mec_spitzbergensis
Pla_sp
Pla_angustata
Pla_horribilis
Rar_minutissimus
Not_pallidoides
Pil_glacialis
Pil_islandicus
Acu_antarcticus
Acu_mariae
Mix_saracenus
Mic_bertolanii
Mic_cf_bertolanii
Cry_antiarktos
Cry_kaczmareki
Heb_conjungens
Heb_ryani
Ram_oberhaeuseri
Ram_varieornatus
Apo_confusus
Dor_flavus
Dor_macrodon
Gre_cryophilus

Gre_granulifer
Psb_kathmanae
Psb_megalonyx
Thu_ruffoi
Thu_stephaniae
Hab_arcturulius
Hab_crispae
Hap_utahensis
Hex_micronyx
Das_improvisus
Din_papillifera
Din_sattleri
Ere_alicatai
Ere_sp_nov
Fra_verrucosus
Iso_arbiter
Iso_prosostomus
Urs_lunulatus
Urs_pappi
Rjd_frigidus
Ado_cf_granulatus
Ado_granulatus
Cre_crenulatus
Cre_ruhesteini
Mac_pallari
Mac_shonaicus
Meb_harmsworthi
Meb_philippinicus
Min_intermedius
Min_ioculator
Pam_areolatus
Pam_richtersi
Sis_spectabilis
Ten_voronkovi
Ten_zandrae
Xer_gretae
Xer_pseudohufelandi
Dac_parthenogeneticus
Dac_selenicus
Mur_cf_pullari
Pmy_dianeae
Dia_islandicus
Ric_coronifer
Ric_ziemowiti

List C

Cal_ornatus
Cal_sp
Dip_higginsi
Dip_puniceum
Kar_gregorii
Kop_nicolae
Bor_zetlandicus
Crb_klebelsbergi
Hys_dujardini
Hys_exemplaris
Adr_belgicae
Adr_scoticum
Ard_tenue
Ard_wuyingensis
Ast_bartosi
Ast_sp
Ast_trinacriae
Gui_prorsirostre
Ins_orientalis
Ita_serratulum
Mec_revelata
Mec_spitzbergensis
Pla_sp
Pla_angustata
Pla_horribilis
Rar_minutissimus
Not_pallidoides
Pil_glacialis
Pil_islandicus
Acu_antarcticus
Acu_mariae
Mix_saracenus
Mic_bertolanii
Mic_cf_bertolanii
Cry_antiarktos
Cry_kaczmareki
Heb_conjungens
Heb_ryani
Ram_oberhaeuseri
Ram_varieornatus

List D

Dip_higginsi
Dip_puniceum
Kar_gregorii
Kop_nicolae
Bor_zetlandicus
Crb_klebelsbergi
Hys_dujardini
Hys_exemplaris
Adr_belgicae
Adr_scoticum
Ard_tenue
Ard_wuyingensis
Ast_bartosi
Ast_sp
Ast_trinacriae
Gui_prorsirostre
Ins_orientalis
Ita_serratulum
Mec_revelata
Mec_spitzbergensis
Pla_sp
Pla_angustata
Pla_horribilis
Rar_minutissimus
Not_pallidoides
Pil_glacialis
Pil_islandicus

Table S4. Measurements of selected characters of *Beorn leggi* from Canadian amber. Measured at the ventral view (*) and dorsal view (†) of the specimen. *br*, the ratio of the secondary claw branch length to the primary claw branch length

CHARACTER	µm	*br*
Body length*	**309**	
Claw 2 right†		
External basal section	**6.8**	**-**
External primary branch	**16.2**	***55.5%***
External secondary branch	**9.0**	**-**
Claw 4 left*		
Anterior basal section	**7.7**	**-**
Anterior primary branch	**7.7**	***76.6%***
Anterior secondary branch	**5.9**	**-**
Posterior basal section	**9.8**	**-**
Posterior primary branch	**14.0**	***67.1%***
Posterior secondary branch	**9.4**	**-**

Table S5. Measurements of selected characters of *Aerobius dactylus* gen. et sp. nov from Canadian amber. Measured at the ventral view of the specimen. *br*, the ratio of the secondary claw branch length to the primary claw branch length. Measurements highlighted in red are only the minimum lengths due to either the claw was not fully extended due to preservation (in claw 2) or the intersection of the primary and secondary branches were not visible (in claws 4)

CHARACTER	µm	*br*
Body length	**100**	
Claw 2 right		
External basal section	**4.2**	**-**
External primary branch	**3.8**	**-**
External secondary branch	**4.9**	**-**
Claw 3 left		
External basal section	**3.8**	**-**
External primary branch	**7.1**	***66.2%***
External secondary branch	**4.7**	**-**
Claw 4 right		
Posterior primary branch	**8.9**	**-**
Claw 4 left		
Posterior primary branch	**11.0**	**-**

Table S6. Kolmogorov-Smirnov test between the posterior (with data) and prior (without data) distributions obtained in BEAST analyses.

Clade	D	p-value
Crown-group Tardigrade	0.092827	<2.2e-16
Crown-group Eutardigrada	0.31468	<2.2e-16
Crown-group Parachela	0.81035	<2.2e-16
Crown-group Heterotardigrada	0.338	<2.2e-16
Crown-group Echiniscoidea	0.37242	<2.2e-16
Oreellidae-Echiniscidae	0.59338	<2.2e-16

Table S7. Genome assembly statistics of *Actinarctus doryphorus*

Statistics	Pre-contamination removal		1st round of contamination removal		2nd round of contamination removal	
	Pre-1000 bp removal	Post-1000 bp removal	Pre-1000 bp removal	Post-1000 bp removal	Pre-1000 bp removal	Post-1000 bp removal
Genome size/total scaffold length	50,618,230	32,035,376	41,392,032	19,718,029	39,450,773	20,292,635
Scaffold number	39998	12929	43363	11514	39352	11394
GC%	50.04	50.16	49.71	49.46	49.74	49.55
Longest scaffold length	274916	274916	39473	39473	37354	37354
N50	1362	2717	964	1649	1023	1741

Table S8. BUSCO statistics of the gene prediction from the *Actinarctus doryphorus* genome assembly. Highlighted in red is the total Complete BUSCO scores.

BUSCOs		vs Eukaryota database	Vs Metazoa database
Complete	Total	23 (9.0%)	89 (9.4%)
	Single-copy	12 (4.7%)	57 (6.0%)
	Duplicated	11 (4.3%)	32 (3.4%)
Fragmented		30 (11.8%)	93 (9.7%)
Missing		202 (79.2%)	772 (80.9%)
Total		255	954

References

1. Mapalo, M. A., Robin, N., Boudinot, B. E., Ortega-Hernández, J. & Barden, P. A tardigrade in Dominican amber. *Proc. R. Soc. B Biol. Sci.* **288**, (2021).
2. Bertolani, R. *et al.* Phylogeny of Eutardigrada: New molecular data and their morphological support lead to the identification of new evolutionary lineages. *Mol. Phylogenet. Evol.* **76**, 110–126 (2014).
3. Guil, N., Jørgensen, A. & Kristensen, R. An upgraded comprehensive multilocus phylogeny of the Tardigrada tree of life. *Zool. Scr.* **48**, 120–137 (2019).
4. Katoh, K. & Standley, D. M. MAFFT multiple sequence alignment software version 7: Improvements in performance and usability. *Mol. Biol. Evol.* **30**, 772–780 (2013).
5. Larsson, A. AliView: A fast and lightweight alignment viewer and editor for large datasets. *Bioinformatics* **30**, 3276–3278 (2014).
6. Gouy, M., Tannier, E., Comte, N. & Parsons, D. P. Seaview Version 5: A multiplatform software for multiple sequence alignment, molecular phylogenetic analyses, and tree reconciliation. in *Multiple Sequence Alignment: Methods and Protocols, Methods in Molecular Biology* (ed. Katoh, K.) vol. 1656 241–260 (Humana, 2021).
7. Ronquist, F. *et al.* Mrbayes 3.2: Efficient Bayesian phylogenetic inference and model choice across a large model space. *Syst. Biol.* **61**, 539–542 (2012).
8. Lewis, P. O. A likelihood approach to estimating phylogeny from discrete morphological character data. *Syst. Biol.* **50**, 913–925 (2001).
9. Lanfear, R., Frandsen, P. B., Wright, A. M., Senfeld, T. & Calcott, B. Partitionfinder 2: New methods for selecting partitioned models of evolution for molecular and morphological phylogenetic analyses. *Mol. Biol. Evol.* **34**, 772–773 (2017).
10. Vecchi, M. *et al.* Expanding *Acutuncus*: Phylogenetics and morphological analyses reveal a considerably wider distribution for this tardigrade genus. *Mol. Phylogenet. Evol.* **180**, 107707 (2023).
11. Mapalo, M. A. *et al.* The unique antimicrobial recognition and signaling pathways in tardigrades with a comparison across ecdysozoa. *G3 Genes|Genomes|Genetics* **10**, 1137–1148 (2020).
12. Howard, R. J. *et al.* The Ediacaran origin of Ecdysozoa: integrating fossil and phylogenomic data. *J. Geol. Soc. London.* **179**, (2022).
13. Howard, R. J. *et al.* A tube-dwelling early Cambrian lobopodian. *Curr. Biol.* **30**, 1529-1536.e2 (2020).
14. Bankevich, A. *et al.* SPAdes: A new genome assembly algorithm and its applications to single-cell sequencing. *J. Comput. Biol.* **19**, 455–477 (2012).
15. Gurevich, A., Saveliev, V., Vyahhi, N. & Tesler, G. QUAST: Quality assessment tool for genome assemblies. *Bioinformatics* **29**, 1072–1075 (2013).
16. Laetsch, D. R. & Blaxter, M. L. BlobTools: Interrogation of genome assemblies. *F1000Research* **6**, 1287 (2017).
17. Stanke, M. *et al.* AUGUSTUS: Ab initio prediction of alternative transcripts. *Nucleic Acids Res.* **34**, 435–439 (2006).
18. Li, W. & Godzik, A. Cd-hit: A fast program for clustering and comparing large sets of protein or nucleotide sequences. *Bioinformatics* **22**, 1658–1659 (2006).
19. Manni, M., Berkeley, M. R., Seppey, M., Simão, F. A. & Zdobnov, E. M. BUSCO update: Novel and streamlined workflows along with broader and deeper phylogenetic coverage for scoring of eukaryotic, prokaryotic, and viral genomes. *Mol. Biol. Evol.* **38**, 4647–4654

(2021).
20. Train, C. M., Glover, N. M., Gonnet, G. H., Altenhoff, A. M. & Dessimoz, C. Orthologous Matrix (OMA) algorithm 2.0: More robust to asymmetric evolutionary rates and more scalable hierarchical orthologous group inference. *Bioinformatics* **33**, i75–i82 (2017).
21. Schwentner, M., Richter, S., Rogers, D. C. & Giribet, G. Tetraconatan phylogeny with special focus on Malacostraca and Branchiopoda: Highlighting the strength of taxon-specific matrices in phylogenomics. *Proc. R. Soc. B Biol. Sci.* **285**, (2018).
22. Smith, S. A. & Dunn, C. W. Phyutility: A phyloinformatics tool for trees, alignments and molecular data. *Bioinformatics* **24**, 715–716 (2008).
23. Nguyen, L. T., Schmidt, H. A., Von Haeseler, A. & Minh, B. Q. IQ-TREE: A fast and effective stochastic algorithm for estimating maximum-likelihood phylogenies. *Mol. Biol. Evol.* **32**, 268–274 (2015).
24. Mirarab, S. & Warnow, T. ASTRAL-II: Coalescent-based species tree estimation with many hundreds of taxa and thousands of genes. *Bioinformatics* **31**, i44–i52 (2015).
25. Reis, M. Dos & Yang, Z. Approximate likelihood calculation on a phylogeny for Bayesian Estimation of Divergence Times. *Mol. Biol. Evol.* **28**, 2161–2172 (2011).
26. Yang, Z. PAML 4: Phylogenetic analysis by maximum likelihood. *Mol. Biol. Evol.* **24**, 1586–1591 (2007).
27. Bartels, P. J., Apodaca, J. J., Mora, C. & Nelson, D. R. A global biodiversity estimate of a poorly known taxon: Phylum Tardigrada. *Zool. J. Linn. Soc.* **178**, 730–736 (2016).
28. Bouckaert, R. R. *et al.* BEAST 2.5: An advanced software platform for Bayesian evolutionary analysis. *PLoS Comput. Biol.* **15**, 1–28 (2019).
29. Bouckaert, R. R. & Drummond, A. J. bModelTest: Bayesian phylogenetic site model averaging and model comparison. *BMC Evol. Biol.* **17**, 1–11 (2017).
30. Rambaut, A., Drummond, A. J., Xie, D., Baele, G. & Suchard, M. A. Posterior summarization in Bayesian phylogenetics using Tracer 1.7. *Syst. Biol.* **67**, 901–904 (2018).
31. Dòyere, L. M. F. Memoire sur les Tardigrades. *Ann. des Sci. Nat. Paris, Ser. 2* **14**, 269–362 (1840).
32. Richters, F. Tardigrada. in *Handbuch der Zoologie 3* (eds. Kükenthal, W. & Krumbach, T.) 1–68 (Walter de Gruyter & Co., 1926).
33. Schuster, R. O., Nelson, D. R., Grigarick, A. A. & Christenberry, D. Systematic criteria of the Eutardigrada. *Trans. Am. Microsc. Soc.* **99**, 284–303 (1980).
34. Pilato, G. Evoluzione e nuova sistemazione degli eutardigrada. *Bolletino di Zool.* **36**, 327–345 (1969).
35. Marley, N. J., Mcinnes, S. J. & Sands, C. J. Phylum Tardigrada: A re-evaluation of the Parachela. *Zootaxa* **64**, 51–64 (2011).
36. Cooper, K. W. The first fossil tardigrade: *Beorn leggi* Cooper, from Cretaceous amber. *Psyche (Stuttg).* **71**, 41–48 (1964).
37. Pilato, G. *Mixibius*, nuovo genere di Hypsibiidae (Eutardigrada). *Animalia* **19**, 121–125 (1992).
38. Pilato, G. & Binda, M. G. Definition of families, subfamilies, genera and subgenera of the Eutardigrada and keys to their identification. *Zootaxa* **2404**, 1–54 (2010).
39. Gąsiorek, P., Morek, W., Stec, D., Blagden, B. & Michalczyk, Ł. Revisiting Calohypsibiidae and Microhypsibiidae: *Fractonotus* Pilato, 1998 and its phylogenetic

position within Isohypsibiidae (Eutardigrada: Parachela). *Zoosystema* **41**, 71–89 (2019).
40. Stec, D., Morek, W., Gąsiorek, P. & Michalczyk, Ł. Unmasking hidden species diversity within the *Ramazzottius oberhaeuseri* complex, with an integrative redescription of the nominal species for the family Ramazzottiidae (Tardigrada: Eutardigrada: Parachela). *Syst. Biodivers.* **16**, 357–376 (2018).
41. Kaczmarek, Ł., Bartylak, T. & Roszkowska, M. Two new genera of long clawed Isohypsibioidea Guil, Jørgensen & Kristensen, 2019. *Zootaxa* **4729**, 293–299 (2020).
42. Perry, E., Miller, W. R. & Kaczmarek, Ł. Recommended abbreviations for the names of genera of the phylum Tardigrada. *Zootaxa* **4608**, 145–154 (2019).
43. Perry, E., Miller, W. R. & Kaczmarek, Ł. Additional recommended abbreviations for the names of genera of the phylum Tardigrada. *Zootaxa* **4981**, 398–400 (2021).
44. Wolfe, J. M., Daley, A. C., Legg, D. A. & Edgecombe, G. D. Fossil calibrations for the arthropod Tree of Life. *Earth-Science Rev.* **160**, 43–110 (2016).
45. Walaszczyk, I. *et al.* The Global Boundary Stratotype Section and Point (GSSP) for the base of the Coniacian Stage (Salzgitter-Salder, Germany) and its auxiliary sections (Słupia Nadbrzeżna, central Poland; Střeleč, Czech Republic; and El Rosario, NE Mexico). *Episodes* **45**, 181–220 (2022).
46. Bertolani, R. & Grimaldi, D. A new eutardigrade (Tardigrada: Milnesiidae) in amber from the Upper Cretaceous (Turonian) of New Jersey. in *Studies on fossils in amber, with particular reference to to the Cretaceous of New Jersey* 103–110 (Backhuys, 2000).
47. Grimaldi, D. A., Nascimbene, P. C. & Penney, D. Raritan (New Jersey) amber. in *Biodiversity of fossils in amber from the major world deposits* (ed. Penney, D.) 167–191 (Siri Scientific Press, 2010).
48. McKellar, R. C., Wolfe, A. P., Tappert, R. & Muehlenbachs, K. Correlation of Grassy Lake and Cedar Lake ambers using infrared spectroscopy, stable isotopes, and palaeoentomology. *Can. J. Earth Sci.* **45**, 1061–1082 (2008).
49. McKellar, R. C. & Wolfe, A. P. Canadian amber. in *Biodiversity of fossils in amber from the major world deposits* (ed. Penney, D.) 96–113 (Siri Scientific Press, 2010).
50. Odin, G. S. & Lamaurelle, M. A. The global Campanian-Maastrichtian stage boundary. *Episodes* **24**, 229–238 (2001).
