# Supplementary material for: Cretaceous amber inclusions illuminate the evolutionary origin of tardigrades
Source: Commun Biol. 2024 Aug 6;7:953. doi: 10.1038/s42003-024-06643-2 (PMC11303527; doi:10.1038/s42003-024-06643-2)
Supplement: Supplementary file 2 — Description of additional supplementary files [file 42003_2024_6643_MOESM2_ESM.pdf]

Description of Additional Supplementary Files

File name: Data S1

Description: Morphological characters used for the total-evidence phylogenetic analysis

File name: Data S2

Description: Character matrix used for the totalevidence phylogenetic analysis

File name: Data S3

Description: 18S alignment used for the totalevidence phylogenetic analysis

File name: Data S4

Description: Translated transcriptome alignment used for MCMCtree analaysis

File name: Data S5

Description: Results of the MCMCTree runs andconvergence test

File name: Data S6

Description: 18S and 28S rRNA Genbank Accession numbers used for the divergencetime estimation analyses

File name: Data S7

Description: Concatenated 18S and 28S rRNA sequences used for the divergence time estimation analyses
