# Supplementary material for: Cretaceous amber inclusions illuminate the evolutionary origin of tardigrades
Source: Commun Biol. 2024 Aug 6;7:953. doi: 10.1038/s42003-024-06643-2 (PMC11303527; doi:10.1038/s42003-024-06643-2)
Supplement: Supplementary file 3 — Data S1: Morphological characters used for the total - evidence phylogenetic analysis [file 42003_2024_6643_MOESM3_ESM.docx]

1. **BODY SURFACE**
2. Peribuccal papillae

0 – absent

1 – present

1. Dorsal cuticle protuberances

0 – absent

1 – present (gibbosities, plaques, tubercles)

1. Elliptical organ

0 – absent

1 – present

1. **CLAWS**
2. ***Claws I-III***
3. Primary branch and secondary branch connection in claws in legs 1-3

0 – not connected

1 – connected

1. Claws 1-3 symmetry of primary and secondary branches with respect to the median plane of the leg

0 – symmetrical

1 – asymmetrical

1. Shape of external and internal claws 1-3

0 – similar

1 – not similar

1. Clear division (septa) of primary branch, secondary branch, and basal section

0 – absent

1 – present

1. External claw primary branch connection to the basal section

0 – direct

1 – with a discernible flexible connection

1. Type of flexible connection of the external claw primary branch

0 – thick, cuticularized

1 – thin, not entirely cuticularized

1. Angular insertion of the external claw secondary branch to the basal section

0 – not perpendicular

1 – perpendicular

1. External claw basal section subdivided into stem/peduncle and distal section

0 – absent

1 – present

1. Internal claw basal section subdivided into stem/peduncle and distal section

0 – absent

1 – present

1. Claws 1-3 base extension

0 – absent

1 – present

1. Type of claws 1-3 base extension

0 – basal thickening

1 – pseudolunules

2 – lunules

1. ***Claws IV***
2. Primary branch and secondary branch connection in claws in legs 4

0 – not connected

1 – connected

1. Claw 4 symmetry of primary and secondary branches with respect to the median plane of the leg

0 – symmetrical

1 – asymmetrical

1. Shape of posterior and anterior claws (claw IV)

0 – similar

1 – not similar

1. Clear division (septa) of primary branch, secondary branch, and basal section

0 – absent

1 – present

1. Posterior claw primary branch connection to the basal section (claw IV)

0 – direct

1 – with a flexible connection

1. Type of flexible connection of the external claw primary branch

0 – thick, cuticularized

1 – thin, not entirely cuticularized

1. Angular insertion of the posterior claw secondary branch to the basal section (claw IV)

0 – not perpendicular

1 – perpendicular

1. Posterior claw basal section subdivided into stem/peduncle and distal section

0 – absent

1 – present

1. Anterior claw basal section subdivided into stem/peduncle and distal section

0 – absent

1 – present

1. Claws 4 base extension

0 – absent

1 – present

1. Type of claws 4 base extension

0 – basal thickening

1 – pseudolunules

2 – lunules

1. **BUCCO-PHARYNGEAL APPARATUS**
2. Peribuccal structure

0 – absent

1 – present

1. Type of peribuccal structure

0 – peribuccal lamellae

1 – peribuccal lamina/velum

1. Dorsal apophysis of the insertion of the stylet muscle (AISM)

0 – absent

1 – present

1. Hook-shaped structure in dorsal apophysis

0 – absent

1 – present

1. Hook-shaped structure in ventral apophysis

0 – absent

1 – present

1. Ventral AISM and dorsal AISM symmetry

0 – asymmetrical

1 – symmetrical

1. Macroplacoids

0 – absent

1 – present

1. Type of macroplacoids

0 – one undivided macroplacoid

1 – divided macroplacoids

1. Pharyngeal apophyses

0 – absent

1 – present

1. Microplacoid

0 – absent

1 – present

1. **EGG MORPHOLOGY**
2. Egg ornamentation

0 – absent/smooth

1 – present

**REFERENCES**

Bertolani, R., R. Guidetti, T. Marchioro, T. Altiero, L. Rebecchi *et al.*, 2014 Phylogeny of Eutardigrada: New molecular data and their morphological support lead to the identification of new evolutionary lineages. Mol. Phylogenet. Evol. 76: 110–126.

Bertolani, R., and G. Pilato, 1988 Struttura delle unghie nei Macrobiotidae e descrizione di *Murrayon* n. gen. (Eutardigrada). Animalia 15: 17–24.

Bertolani, R., and L. Rebecchi, 1993 A revision of the *Macrobiotus hufelandi* group (Tardigrada, Macrobiotidae), with some observations on the taxonomic characters of eutardigrades. Zool. Scr. 22: 127–152.

Bertolani, R., L. Rebecchi, I. Giovannini, and M. Cesari, 2011 DNA barcoding and integrative taxonomy of *Macrobiotus hufelandi* C.A.S. Schultze 1834, the first tardigrade species to be described, and some related species. Zootaxa 36: 19–36.

Binda, M. G., and G. Pilato, 1969 Tardigradi muscicoli dell’isola di Ustica (Sicilia), con descrizione di due specie nuove. Boll. delle sedute dell’Accademia Gioenia di Sci. Nat. Catania - Ser. 4 10: 170–180.

Gąsiorek P., W. Morek, D. Stec, B. Blagden, and Ł. Michalczyk, 2019 Revisiting Calohypsibiidae and Microhypsibiidae: *Fractonotus* Pilato, 1998 and its phylogenetic position within Isohypsibiidae (Eutardigrada: Parachela). Zoosystema 41: 71–89.

Gąsiorek, P., D. Stec, W. Morek, and Ł. Michalczyk, 2018 An integrative redescription of *Hypsibius dujardini* (Doyère, 1840), the nominal taxon for Hypsibioidea (Tardigrada: Eutardigrada). Zootaxa 4415: 45–75.

Gąsiorek, P., D. Stec, W. Morek, and Ł. Michalczyk, 2019 Deceptive conservatism of claws: Distinct phyletic lineages concealed within Isohypsibioidea (Eutardigrada) revealed by molecular and morphological evidence. Contrib. to Zool. 88: 78–132.

Guidetti, R., T. Altiero, T. Marchioro, L. S. Amadè, A. M. Avdonina *et al.*, 2012 Form and function of the feeding apparatus in Eutardigrada (Tardigrada). Zoomorphology 131: 127–148.

Kaczmarek, Ł., and Ł. Michalczyk, 2017 The *Macrobiotus* *hufelandi* group (Tardigrada) revisited. Zootaxa 4363: 101–123.

Kristensen, R. M., 1982 New aberrant Eutardigrades from homothermic springs on Disko Island, West Greenland, pp. 203–220 in *Proceedings of the Third International Symposium on Tardigrada*, edited by D. R. Nelson. East Tennessee State University Press, Johnson City, Tennessee.

Kristensen, R. M., 1983 The first record of cyclomorphosis in Tardigrada based on a new genus and species from Arctic meiobenthos. J. Zool. Syst. Evol. Res. 20: 249–270.

Maucci, W., and G. Ramazzotti, 1981 *Adorybiotus* gen. nov.: Nuova posizione sistematica *Macrobiotus granulatus* Richters, 1903 e per *Macrobiotus* *coronifer* Richters, 1903 (Tardigrada, Macrobiotidae). Mem. dell’Instituto Ital. di Idrobiol. 39: 153–159.

Michalczyk, Ł., W. Wełnicz, M. Frohme, and Ł. Kaczmarek, 2012 Redescriptions of three *Milnesium* Doyère, 1840 taxa (Tardigrada: Eutardigrada: Milnesiidae), including the nominal species for the genus. Zootaxa 20: 1–20.

Murray, J., 1907 Scottish Tardigrada, collected by the Lake Survey. Trans. R. Soc. Edinburgh 24: 641–668.

Pilato, G., 1969 Su un interessante Tardigrado esapodo delle dune costiere siciliane: *Hexapodibius micronyx* n. gen. n. sp. Boll. delle sedute dell’Accademia Gioenia di Sci. Nat. Catania - Ser. 4 9: 619–622.

Pilato, G., 2013 The taxonomic value of the structures for the insertion of the stylet muscles in the Eutardigrada, and description of a new genus. Zootaxa 3721: 365–378.

Pilato, G., and M. G. Binda, 2010 Definition of families, subfamilies, genera and subgenera of the Eutardigrada, and keys to their identification. Zootaxa 54: 1–54.

Pilato, G., and M. G. Binda, 1987 *Richtersia*, nuovo genere di Macrobiotidae, e nuova definizione di *Adorybiotus* Maucci e Ramazzotti, 1981 (Eutardigrada). Animalia 14: 147–152.

Richters, F., 1904 Arktische Tardigraden. Fauna Arctica 3: 494–508.

Richters, F., 1900 Beiträge zur Kenntnis der Fauna der Umggegend von Frankfurt a. M. Bericht der Senckenbergischen Naturforschenden gesellschaft Frankfurt am Main 21–44.

Smykla, J., Ł. Kaczmarek, K. Huzarska, and Ł. Michalczyk, 2011 The first record of a rare marine tardigrade, *Halobiotus crispae* Kristensen, 1982 (Eutardigrada: Hypsibiidae), from the Svalbard Archipelago. Polar Biol. 34: 1243–1247.

Stec, D., Ł. Krzywański, K. Arakawa, and Ł. Michalczyk, 2020a A new redescription of *Richtersius* *coronifer*, supported by transcriptome, provides resources for describing concealed species diversity within the monotypic genus *Richtersius* (Eutardigrada). Zool. Lett. 6: 1–25.

Stec, D., W. Morek, P. Gąsiorek, and Ł. Michalczyk, 2018 Unmasking hidden species diversity within the *Ramazzottius oberhaeuseri* complex, with an integrative redescription of the nominal species for the family Ramazzottiidae (Tardigrada: Eutardigrada: Parachela). Syst. Biodivers. 1–20.

Stec, D., M. Vecchi, W. Maciejowski, and Ł. Michalczyk, 2020b Resolving the systematics of Richtersiidae by multilocus phylogeny and an integrative redescription of the nominal species for the genus *Crenubiotus* (Tardigrada). Sci. Rep. 10: 1–20.

Thulin, G., 1911 Beiträge zur Kenntnis der Tardigradenfauna Schwedens. Ark. för Zool. 7: 1–60.

Thulin, G., 1928 Über die Phylogenie und das System der Tardigraden. Hereditas 11: 207–266.

Trygvadóttir, B. V., and R. M. Kristensen, 2011 Eohypsibiidae (Eutardigrada, Tardigrada) from the Faroe Islands with the description of a new genus containing three new species. Zootaxa 62: 39–62.
