# Supplementary material for: Cretaceous amber inclusions illuminate the evolutionary origin of tardigrades
Source: Commun Biol. 2024 Aug 6;7:953. doi: 10.1038/s42003-024-06643-2 (PMC11303527; doi:10.1038/s42003-024-06643-2)
Supplement: Supplementary file 10 — Reporting Summary [file 42003_2024_6643_MOESM10_ESM.pdf]

nature portfolio

Corresponding author(s): Marc Mapalo

Last updated by author(s): Jul 2, 2024

Reporting Summary

Nature Portfolio wishes to improve the reproducibility of the work that we publish. This form provides structure for consistency and transparency in reporting. For further information on Nature Portfolio policies, see our Editorial Policies and the Editorial Policy Checklist.

Statistics

For all statistical analyses, confirm that the following items are present in the figure legend, table legend, main text, or Methods section.

n/a	Confirmed	
☐	☒	The exact sample size (*n*) for each experimental group/condition, given as a discrete number and unit of measurement
☐	☒	A statement on whether measurements were taken from distinct samples or whether the same sample was measured repeatedly
☐	☒	The statistical test(s) used AND whether they are one- or two-sided<br>*Only common tests should be described solely by name; describe more complex techniques in the Methods section.*
☒	☐	A description of all covariates tested
☒	☐	A description of any assumptions or corrections, such as tests of normality and adjustment for multiple comparisons
☐	☒	A full description of the statistical parameters including central tendency (e.g. means) or other basic estimates (e.g. regression coefficient) AND variation (e.g. standard deviation) or associated estimates of uncertainty (e.g. confidence intervals)
☐	☒	For null hypothesis testing, the test statistic (e.g. *F*, *t*, *r*) with confidence intervals, effect sizes, degrees of freedom and *P* value noted<br>*Give P values as exact values whenever suitable.*
☐	☒	For Bayesian analysis, information on the choice of priors and Markov chain Monte Carlo settings
☒	☐	For hierarchical and complex designs, identification of the appropriate level for tests and full reporting of outcomes
☒	☐	Estimates of effect sizes (e.g. Cohen's *d*, Pearson's *r*), indicating how they were calculated

Our web collection on statistics for biologists contains articles on many of the points above.

Software and code

Policy information about availability of computer code

Data collection	All data obtained from the public repository are listed in the Supplementary Material, including links where they were obtained.
Data analysis	All scripts used for data analysis and visualization are included in the Supplementary Material

For manuscripts utilizing custom algorithms or software that are central to the research but not yet described in published literature, software must be made available to editors and reviewers. We strongly encourage code deposition in a community repository (e.g. GitHub). See the Nature Portfolio guidelines for submitting code & software for further information.

Data

Policy information about availability of data

All manuscripts must include a data availability statement. This statement should provide the following information, where applicable:

- Accession codes, unique identifiers, or web links for publicly available datasets
- A description of any restrictions on data availability
- For clinical datasets or third party data, please ensure that the statement adheres to our policy

All data files supporting the findings of this study are available within the published manuscript and the Supplementary Materials (i.e., 18S and 28S rRNA accession numbers, morphological character list, sequence alignments, and MCMCTree runs and convergence tests). All raw files from the phylogenetic analysis (i.e., t, p, and tree files from MrBayes, output tree from ASTRAL and IQTree, mcmc files from MCMCTree, and tre and log files from BEAST), RScripts used, and assembled Actinarctus doryphorus genome are available on Dryad Digital Repository (https://doi.org/10.5061/dryad.s1rn8pkfx). Character matrix is also available on

MorphoBank (http://morphobank.org/permalink/?P4855). Zoobank registration for Aerobius dactylus:http://www.zoobank.org/urn:lsid:zoobank.org:pub:E407CAA2-4928-4670-AB33-F4E5E9E4A589. All data can also be requested from the corresponding authors.

Human research participants

Policy information about studies involving human research participants and Sex and Gender in Research.

Reporting on sex and gender	N/A
Population characteristics	N/A
Recruitment	N/A
Ethics oversight	N/A

Note that full information on the approval of the study protocol must also be provided in the manuscript.

Field-specific reporting

Please select the one below that is the best fit for your research. If you are not sure, read the appropriate sections before making your selection.

☐ Life sciences ☐ Behavioural & social sciences ☒ Ecological, evolutionary & environmental sciences

For a reference copy of the document with all sections, see nature.com/documents/nr-reporting-summary-flat.pdf

Ecological, evolutionary & environmental sciences study design

All studies must disclose on these points even when the disclosure is negative.

Study description	The study re-described two fossil tardigrades by producing high-quality images using confocal fluorescence microscopy. This allowed the description of one new species and the resolution of the phylogenetic relationships of these fossils with respect to extant tardigrades. This new information was allowed the use of new calibration strategies to calculate divergence time estimates and to provide timings of important macroevolutionary events in the tardigrade lineage
Research sample	The two fossils are embedded in a Cretaceous-aged amber and deposited in the Museum of Comparative Zoology in Harvard University. They were the first tardigrade fossils to be described back in 1964 and constitute 50% of the known tardigrade fossils in the entire world
Sampling strategy	N/A
Data collection	Confocal images were obtained using microscopes from the Harvard Center for Biological Imaging. All sequences used for the phylogenies and molecular dating analyses were obtained in public repositories. Exact details are provided in the Supplementary Materials
Timing and spatial scale	N/A
Data exclusions	N/A
Reproducibility	All necessary data to enable reproducibility of the results are in the Supplementary Materials
Randomization	N/A
Blinding	N/A

Did the study involve field work? ☐ Yes ☒ No

Reporting for specific materials, systems and methods

We require information from authors about some types of materials, experimental systems and methods used in many studies. Here, indicate whether each material, system or method listed is relevant to your study. If you are not sure if a list item applies to your research, read the appropriate section before selecting a response.

Materials & experimental systems

n/a	Involved in the study
☒	☐ Antibodies
☒	☐ Eukaryotic cell lines
☐	☒ Palaeontology and archaeology
☒	☐ Animals and other organisms
☒	☐ Clinical data
☒	☐ Dual use research of concern

Methods

n/a	Involved in the study
☒	☐ ChIP-seq
☒	☐ Flow cytometry
☒	☐ MRI-based neuroimaging

Palaeontology and Archaeology

Specimen provenance: The studied amber material was part of secondary deposits collected by William M. Legg in 1940 along beaches near the entrance of the Saskatchewan River into Cedar Lake, southeast of The Pas, Manitoba, Canada.

Specimen deposition: The fossils are housed at the Entomology Collection at the Museum of Comparative Zoology (MCZ), Harvard University (MCZ PALE-5213 and PALE-45862).

Dating methods: N/A

☐ Tick this box to confirm that the raw and calibrated dates are available in the paper or in Supplementary Information.

Ethics oversight: No ethical approval or guidance was required because we are using fossil samples deposited in a publicly-accessible museum.

Note that full information on the approval of the study protocol must also be provided in the manuscript.
